# Simulating human exposure to indoor airborne microplastics using a Breathing Thermal Manikin

**DOI:** 10.1038/s41598-019-45054-w

**Published:** 2019-06-17

**Authors:** Alvise Vianello, Rasmus Lund Jensen, Li Liu, Jes Vollertsen

**Affiliations:** 1 Department of Civil Engineering, Aalborg University, Thomas Manns Vej 23, 9220 Aalborg Øst, Denmark; 2 School of Architecture, Tsinghua University, Haidian District, 100084 Beijing, China

**Keywords:** Environmental impact, Infrared spectroscopy, Civil engineering

## Abstract

Humans are potentially exposed to microplastics through food, drink, and air. The first two pathways have received quite some scientific attention, while little is known about the latter. We address the exposure of humans to indoor airborne microplastics using a Breathing Thermal Manikin. Three apartments were investigated, and samples analysed through FPA-µFTIR-Imaging spectroscopy followed by automatic analyses down to 11 µm particle size. All samples were contaminated with microplastics, with concentrations between 1.7 and 16.2 particles m^−3^. Synthetic fragments and fibres accounted, on average, for 4% of the total identified particles, while nonsynthetic particles of protein and cellulose constituted 91% and 4%, respectively. Polyester was the predominant synthetic polymer in all samples (81%), followed by polyethylene (5%), and nylon (3%). Microplastics were typically of smaller size than nonsynthetic particles. As the identified microplastics can be inhaled, these results highlight the potential direct human exposure to microplastic contamination via indoor air.

## Introduction

Microplastics (MP) are present everywhere and have received attention due to their persistent nature^1^ and potential impacts on humans^2^ and the environment^3,4^. Most MP are generated by the breakdown of larger items such as clothings^5^, car tires^6^, and mismanaged urban plastic waste^7^. In the indoor environment, there are many goods, materials, and interior furnishings that can give off plastic fragments due to wear and tear^8^, and it has been argued that these sources are substantially more important for human exposure than MP contained in food and drink^9^. The occurrence, sources, and fate of atmospheric MP in the urban compartments, though, are still poorly documented^10–12^.

A crucial aspect of the presence of atmospheric MP is related to its likelihood of being inhaled and potentially reaching the alveoli of the lungs^13^ (defined as breathable particles). The inhalability of a particle is size and shape dependent, as only the smallest particles below 5 µm and fibrous particles seem to be able to be deposited in the deep lung^13,14^. Even though most of the bigger particles (inhalable particles) are subjected to mucociliary clearance in the upper airways, some of them can escape this mechanism and also be deposited in the deep lung. These particles (especially the longer fibres) tend to avoid clearance^15^ and show extreme durability in physiological fluids, likely persisting and accumulating when breathed in^16^. Previous studies have highlighted the presence of synthetic fibres in the lung tissue of workers in the textile industry^17^, showing cases of respiratory irritation^15,18^. The potential mechanism of toxicity for synthetic particles and microfibers is still not fully explained. Greim *et al*.^19^ suggested that the toxicity could be approximated by the contact between vitreous particles/fibres and cells. They found that this interaction can lead to lung inflammation via intracellular messengers and cytotoxic factors which are released, and then cause secondary genotoxicity due to the continuous formation of reactive oxygen species^19^. Particles caught in the mucus of the lungs and nose can be evacuated by for example coughing, blowing the nose, or sneezing and spat out or swallowed with the mucus. In the latter case, the particles will enter the digestive tract, where they might have impacts similar to MP ingested from food and drinks.

Airborne MP are suspected of carrying micropollutants adsorbed to their hydrophobic surface, especially when related to urban environments where Polycyclic Aromatic Hydrocarbons (PAHs) and metals are produced by various emissions. In addition to the adsorbed pollutants, MP may also contain unreacted monomers, additives, dyes, and pigments which could lead to adverse health effects^13^. Although some research has been done to assess the contribution of MP in indoor and outdoor air^11,10^, there is still a substantial lack of information regarding potential exposure and its associated potential threat.

Airborne microplastic pollution represents a new analytical challenge, and there is an urgent need to reduce the (size) limit of detection in the MP analysis by developing and verifying analytical methods capable of consistently detecting particles and fibres down to a few micrometres. Fourier Transform Infrared Spectroscopy (FTIR) is generally perceived as the most suitable analytical tool for MP analysis^20^. The detection of small particles can be carried out by FPA-µFTIR-Imaging analysis (Focal Plane Array-Fourier Transform-Infrared-micro-spectroscopy), which is, thus far, considered the most promising approach for small MP. It avoids the pre-sorting of MP, hereby providing data unbiased by the analyst^21,22^. To our knowledge, no other study has previously used this technique to investigate microplastics in air samples.

To date, only a few studies have addressed the potential human exposure to airborne microplastics^11,13^. This study aims to present the first data on simulated human exposure to airborne microplastics in indoor environments, collecting air samples with a breathing thermal manikin that simulates human metabolic rate and breathing. Sample analysis was performed using state-of-the-art FPA-µFTIR-Imaging spectroscopy, followed by automated MP detection to provide unbiased qualitative and quantitative data. The present study extends knowledge of indoor airborne MP exposure, composition, and size ranges, providing data on particles (including fibres) of sizes down to 11 µm (major dimension).

## Results

### Monitoring contamination

Three procedural blanks were analysed to monitor potential sources of contamination affecting the analysis. The degree of contamination was normalised against the number of blanks and not the filtered air volume, as the contamination was related to handling the sampling equipment, preparing the sample for analysis, and finally the analysis itself. The results showed a contamination of 7.7 ± 3.8 MP per blank sample. The polymeric composition of the contaminating MP was 43% polyester, 22% nylon (synthetic polyamides), 17% polystyrene, 13% polyethylene, 4% polyurethane. The measured contamination of nonsynthetic materials (protein-based material and cellulose) per blank sample was 111 ± 62 particles for protein-based material and 32 ± 23 for cellulose. Comparing to the obtained air exposure samples, the average contamination was 4.9 ± 3.9% for MP and 4.2 ± 5.7% for nonsynthetic particles, corresponding to an overall contamination of 4.2 ± 5.7%. Results were not corrected for contamination.

### Particle exposure

**Figure 1:**
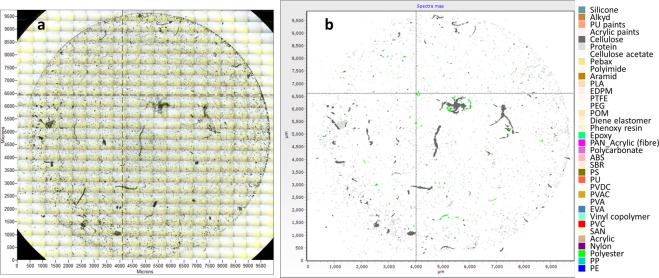
(**a**) Visual stitched image of a sample (L3S3) and (**b**) corresponding MPhunter map. Each polymer group is highlighted by a different colour. Cellulose and protein-based fragments and fibres are shown in grey colours.

**Figure 2:**
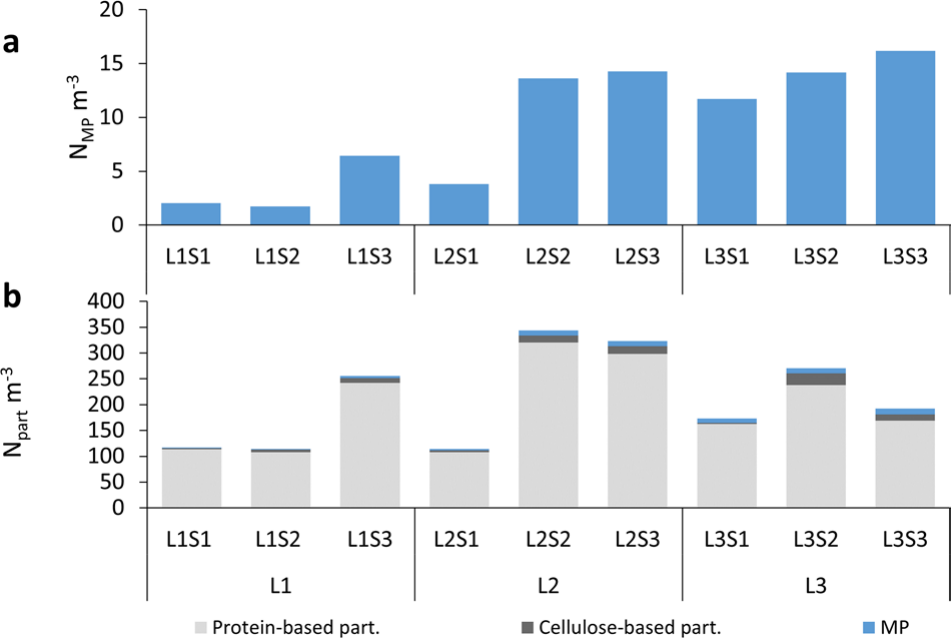
(**a**) Microplastic particle exposure; (**b**) Total particle exposure (MP and nonsynthetic particles) (light grey column – protein-based particles; dark grey column – cellulose-based particles; blue column – MP).

FPA-µFTIR-Imaging combined with automatic particle detection applying the software MPhunter^23^ (SI 1) produced nine particle maps (one per sample). The presence of synthetic and nonsynthetic particles was evaluated by the correlation of each map pixel to a spectral reference database (Fig. 1). All samples revealed the presence of MP as well as nonsynthetic particles (Fig. 2).

The total number of MP inhaled by the manikin over 24 hours reached up to 272 MP (L3S3). The average number of MP inhaled by the manikin per unit volume (N_MP_ m^−3^) over 24 hours was, on average, 9.3 ± 5.8 N_MP_ m^−3^. It ranged from 1.7 N_MP_ m^−3^ at Location 1 (L1), Sample 1 (S1) (L1S1), to 16.2 N_MP_ m^−3^ in L3S3. The MP exposure was highest at Location 3 (14.0 ± 2.2 N_MP_ m^−3^), followed by Location 2 (10.6 ± 5.9 N_MP_ m^−3^) and Location 1 (3.4 ± 2.6 N_MP_ m^−3^) (Fig. 2a). The exposure concentration for nonsynthetic particles was, on average, 205 ± 87 N_part_ m^−3^, ranging from 112 N_part_ m^−3^ in L2S1 to 334 N_part_ m^−3^ in L2S2 (Fig. 2b). Of the three locations, L1 had a nonsynthetic average exposure of 160 ± 79 N_part_ m^−3^, while the concentrations at L2 and L3 were 253 ± 123 N_part_ m^−3^ and 202 ± 51 N_part_ m^−3^, respectively.

### MP and nonsynthetic particles composition

**Figure 3:**
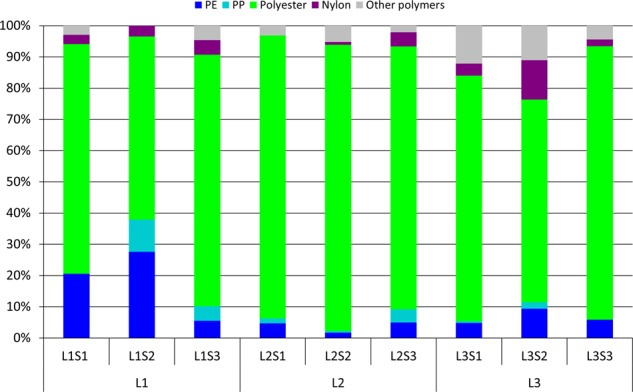
Relative polymer distribution. The category “Other polymers” groups the polymers present in lower percentages (polystyrene - PS, acrylic/acrylates polymers, polyurethane/polyether-urethane - PU, ethylene-propylene-diene-monomer - EPDM, polyvinyl acetate - PVAC, ethylene vinyl acetate - EVA, epoxy resin, phenoxy resin, cellulose acetate and triacetate, polylactic acid - PLA, polycarbonate - PC, acrylic paints, polyurethane paints, alkyd).

The polymer types identified in the samples were quite similar (Fig. 3). The most abundant, among the synthetic polymers, were polyester (59–92%), polyethylene (5–28%), nylon (0–13%), and polypropylene (0.4–10%). The other polymers occurred at lower percentages and were grouped as the sum of polystyrene, acrylic/acrylates polymers, polyurethane/polyether-urethane, ethylene-propylene-diene-monomer, polyvinyl acetate, ethylene vinyl acetate, epoxy resin, phenoxy resin, cellulose acetate and triacetate, polylactic acid, polycarbonate, acrylic paint, polyurethane paint, and alkyd (0–15%) (the percentages are relative to only synthetic particles). Overall, the polymer composition was 81% polyester, 6% polyethylene, 5% nylon, 2% polypropylene, and 6% other polymers. The identified nonsynthetic particles were protein-based and cellulose-based particles. The first type constituted 95% (91–98%) of all nonsynthetic particles, while the latter constituted 5% (2–6%). The FPA-µFTIR-Imaging analysis did not allow discrimination within the nonsynthetic material groups, for example between wool and other protein-based material, or between – for example - cotton and other cellulose-based material. Combining the results for synthetic and nonsynthetic particles (MP, proteins, cellulose), proteins accounted for 91% of the total particle number, while cellulose and MP were both slightly above 4%.

### Particle shape and size measurements

**Figure 4:**
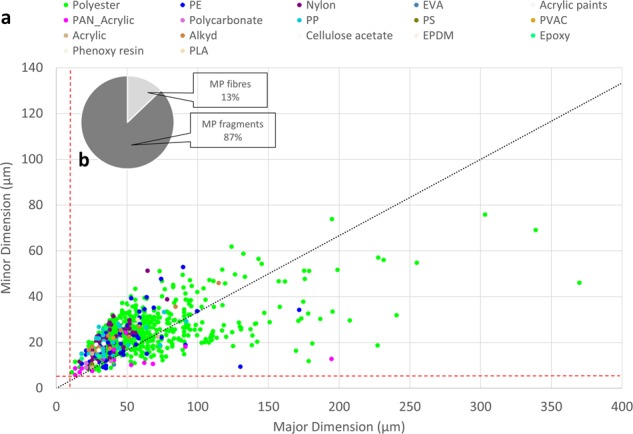
(**a**) MP minor dimension vs major dimension scatter plot. The black dashed line indicates the threshold for fibre classification (length to width ratio of 3); the vertical and horizontal red dashed lines indicate the limit of detection concerning size for major (11 µm) and minor (5.5 µm) dimension (2 × 1 pixels). (**b**) Percentage of MP fibres and fragments for the total analysed samples.

The size and shape of the particles were characterised by two dimensions. This approach was chosen as the MP were mainly irregularly shaped (Fig. 4), and characterising their shape and size with only one dimension was insufficient to distinguish between fibres (length to width ratio > 3) and fragments (length to width ratio ≤ 3). The automated size determination of MPhunter approximates the shape of a particle through an ellipse. The error of this approach was assessed by manually measuring 100 randomly chosen particles of both fragment-like (N = 50) and elongated, fibre-like shapes (N = 50), and comparing the results obtained to the automated determination. Regarding the fragment-like sub-sample, the automatic and manual measurements were not significantly different (major dimension: p = 0.986; minor dimension: p = 0.092), highlighting a good match between automatic and manual measurements. The median value of the automatic and manual determined major dimension of the 50 fragment-like particles was the same (68 µm), while the automatically determined minor dimension was 37 µm, while the manual one was 44 µm. The automatic and manual measurements of the fibre-like particles were not significantly different for the major dimension (p = 0.102), while they differed for the width (p = 3.49e^−06^). In numbers, the automatically determined median length of the 50 randomly selected fibres was 177 µm, while the manual measurement yielded 237 µm. The corresponding widths were 30 µm and 26 µm, respectively. This discrepancy between the manual and automated size determination was deemed acceptable, as it was mainly used to determine if the ratio of the major to minor dimension was above or below 3, and because the error in size determination decreased with a decreasing ratio, i.e. the less elongated a particle was, the better it was described by the automated approach (details are given in SI 1).

**Figure 5:**
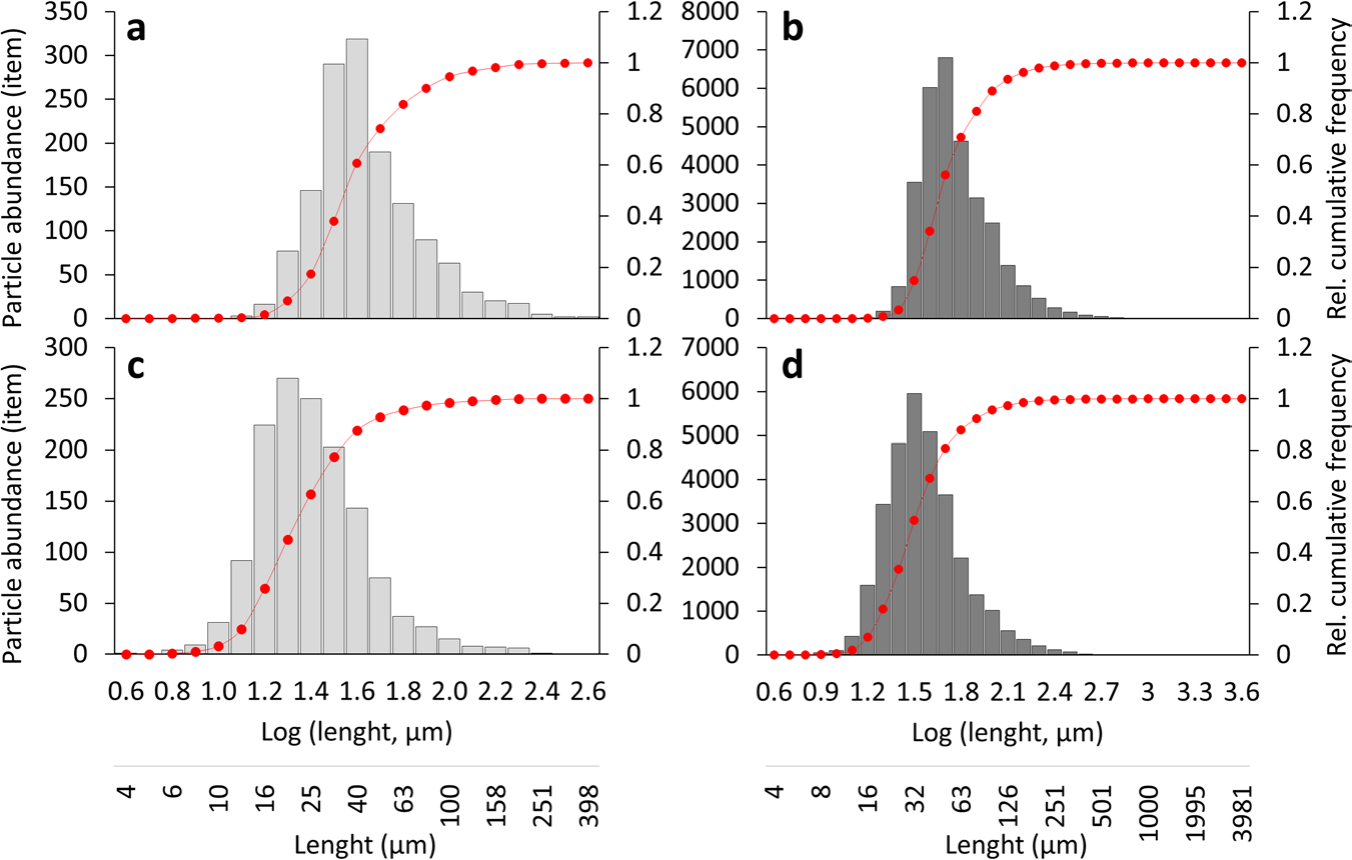
Size distribution for the total amount of MP (**a,c**) and nonsynthetic particles (**b,d**) identified in all analysed samples for major dimension (**a,b**) and minor dimension (**c,d**). Bin intervals were selected as 0.1 on a logarithmic scale. Light and dark grey bars on histograms indicate abundance; the red dotted line is the relative cumulative frequency (secondary axis).

Overall, the median of the length to width ratio of the identified particles was 1.9. The percentage of fibres ranged from 5% in L3S1 to 22%, respectively, in L2S1. Overall, 13% of the identified particles were classified as fibres, while 87% were classified as fragments (Fig. 4). A Shapiro-Wilk test on the distributions of the particle’s major and minor dimension yielded that all size distributions (MP, nonsynthetic, and total particles) were non-normal distributed (p < 0.05). Therefore, median values (D50) were used to describe the data (Table 1). Overall, the size distribution of the identified MP (Fig. 5a,c) had D50s of 36 µm and 21 µm for the major and minor dimension, respectively, while the corresponding D50s for nonsynthetic particles (Fig. 5b,d) were 47 µm and 31 µm.

**Table 1:** Calculated D10, D50, and D90 values relative to the single sample size distributions, the three locations (size distribution obtained from the sum of the three samples from the same location), and the total of the analysed particles (sum of all the particles). Size distribution parameters are displayed both for the major dimension (top value at every sample/location, black coloured digit) and minor dimension (bottom value, italic, bold coloured digit).

Samples	MP			Nonsynthetic		
	D10	D50	D90	D10	D50	D90
L1S1	21	45	98	28	47	99
	***12***	***20***	***39***	***16***	***25***	***42***
L1S2	31	53	87	30	54	138
	***14***	***21***	***36***	***17***	***27***	***49***
L1S3	30	51	121	31	52	121
	***12***	***18***	***30***	***17***	***28***	***50***
L2S1	24	46	105	29	45	92
	***12***	***21***	***37***	***16***	***26***	***42***
L2S2	15	25	43	27	45	101
	***13***	***19***	***29***	***16***	***25***	***44***
L2S3	20	36	79	31	50	115
	***11***	***18***	***30***	***18***	***30***	***49***
L3S1	22	35	69	30	45	80
	***13***	***19***	***31***	***17***	***27***	***40***
L3S2	20	34	75	29	47	112
	***11***	***18***	***30***	***17***	***27***	***47***
L3S3	20	35	79	27	44	84
	***11***	***18***	***30***	***13***	***24***	***39***
**Locations**						
L1	24	41	94	29	51	119
	***17***	***32***	***69***	***19***	***37***	***92***
L2	22	36	82	29	47	106
	***17***	***33***	***78***	***17***	***33***	***79***
L3	21	35	74	28	44	82
	***11***	***18***	***31***	***16***	***26***	***43***
**Total**	21	36	89	29	47	105
	***13***	***21***	***45***	***17***	***31***	***71***

## Discussion

The results highlight the ubiquitous presence of microplastics in inhaled indoor air (Fig. 1). The use of the BTM as a sampling device yields an accurate local airflow at the chosen conditions. Although the simple design of the BTM cannot replicate the complexity of the branching airways of the human lung, the use of the BTM as a sampling device yields an accurate potential dose at the chosen conditions^24^, ensuring a natural mixing of local airstreams, and thus a truer mix for that given situation, than using a standard air sampling device^25^. On average, the measured MP concentration was 9.3 ± 5.8 N_MP_ m^−3^, corresponding well to what Dris *et al*.^11^ reported on indoor air (a median value of 5.4 fibres m^−3^). However, a direct comparison between the studies is problematic, as the analytical approach they applied (manual sorting followed by ATR-FTIR vs µFTIR-Imaging) was different, as was the investigated size range and particle morphology (Dris *et al*. looked solely at fibres down to a major dimension of 50 µm). The highest exposure concentration (16.2 N_MP_ m^−3^) was measured at Location 3 (L3S3), which corresponds to an inhalation rate of 11.3 MP per hour. At such a rate, an average male person doing light activity would potentially inhale up to 272 MP over 24 hours. Cellulose materials were similarly abundant in the inhaled air, and presumably came mainly from cotton and paper products. The most abundant material group, the protein-based materials, most likely almost entirely came from shed skin^26^. Looking solely at the particles of manufactured origin (MP and cellulose), MP accounted, on average, for 50%. Dris *et al*.^11^ reported a slightly different proportion between nonsynthetic and synthetic fibres (67% nonsynthetic and 33% petrochemical). The composition of the inhaled MP (Fig. 3) also differed from the indoor MP composition reported in that study, where polypropylene was the most abundant polymer, while no polyester was found.

In the present study, polyester was by far the most abundant synthetic polymer in all the samples (81%; Fig. 3). The ubiquitous presence of polyester in the inhalable indoor air can be explained by the fact that there are multiple potential sources of polyester fibres and fragments in an indoor environment. Nowadays, most cloths include this type of fibre, as do the majority of the textiles involved in furniture and carpet production. Nylon accounted for 5% of the total identified MP. Although this polymer finds fewer applications in indoor environments than polyester, nylon is still likely to be found in indoor fabrics. Polyethylene and polypropylene accounted for 6% and 2% of the total identified MP, respectively. Even though polyolefin fibres, such as polyethylene and polypropylene, are used for several applications in the textile industry^27^, the reported values were probably influenced by the presence of particles which originated from other sources. While it is possible to account for several sources of fragments and fibres in an indoor environment (carpets, sofas, chairs, etc.) for polypropylene, polyethylene does not find a broad range of applications in the common fibres market, being mostly used for technical textile production^28^ (e.g. high-performance textiles like Dyneema® and Spectra®). Therefore, polyethylene micro-particles probably originated from other sources, like micro-debris fragmenting from packaging materials or other plastic items inside the apartments. Among the polymers identified by FPA-µFTIR-Imaging at a lower percentage (<1%), it is worth mentioning the presence of polyurethane and paint (acrylic and alkyd) micro-particles. Polyurethanes (PU) constitutes a wide group of polymers with a broad range of applications. Some of the chemicals involved in PU production are considered to be harmful substances, and, as for many other additives in plastics, there is the possibility that they could be released in the environment. Moreover, several polyurethanes used in furniture are also treated with flame retardants, of which almost all are considered harmful^29,30^. The potential risk associated with the micro-paint particles could be derived from the organic compounds and heavy metals used as biocides in many paints^31^, as well as the fillers and the pigments^32^, all of which could potentially be released in the environment and could also lead to a direct impact regarding human exposure. The presence of airborne polyurethane and micro-paint particles, and moreover their availability to be inhaled, hence constitutes a potential tread to human health even though they occur at low concentrations.

Besides the ubiquitous exposure to MP pollution, the results also highlight a large variability in the concentration among the samples, both when considering the inter-location variations (58% for MP; 22% for nonsynthetic particles; 23% for total particles) and the intra-location variations (16–77% for MP; 25–49% for nonsynthetic particles; 24–50% for the total particles). The difference in MP concentration between the three apartments was significant (p = 0.041;) when comparing L1 and L3 (p = 0.037), but not when comparing L2 to L1 (p = 0.143) or L3 (p = 0.562) (Fig. 2). L1 also tended to have relatively fewer polyester particles and relatively more polyethylene particles than the other apartments, while L3 tended to have a larger fraction of nylon and “other polymers” compared to L1 and L2. However, the abundance of nonsynthetic particles was comparable between all apartments (p = 0.487). Differences in building materials, furniture, cleaning procedures, and activities among the apartments could explain the inter-location variations of the measured MP exposure concentration. Intra-location variations could be related to activities happening during the sampling, which could have temporarily modified the particle concentration in the indoor air. Sample preparation could be another parameter which influenced the obtained results. While probably only of minor importance, the transfer of the sample from the filtration membrane to the analytical substrate could have caused some loss of particles.

As highlighted by the dots shown below the black dashed line of Fig. 4a, most MP classified as fibres were composed of polyester (87%), followed by polyethylene (6%), polyacrylonitrile (PAN - acrylic fibre, 4%) and polypropylene (1%). The other 2% identified as MP fibres were composed of acrylic polymers, acrylic paints, ethylene vinyl acetate, and polycarbonate. Due to their polymeric composition, these latter particles were probably elongated fragments and not true fibres, as these polymers are not commonly used in textile manufacturing. Surprisingly, no nylon particles were classified as fibres. Although polyester was the most abundant polymer among the synthetic fibrous material, fibres only constituted 13% of the total amont of this polymer. A probable cause is that single fibres might have been entwined or interwoven in fragments of fabric, and so identified as particles. Moreover, polyester sources other than textiles could occur in an indoor environment, as this polymer is also widely used in packaging and plastic items manufacturing. Among the identified polymers, 50% of the PAN particles were classified as fibres, highlighting that this polymer is mainly used in the textile industry. Half of the identified MP were smaller than 50 µm, as shown by the D50 values (Table 1), confirming the presence of small MP (<50 µm) in the air compartment. MP size distributions at the three locations were statistically different for both major and minor dimensions (all p values were below 0.05).

The overall shape of the size distributions when binning particle sizes in intervals of 0.1 on a logarithmic scale (Fig. 5), showed that few particles were present in the larger size bins. The curve then peaked at some size, upon which it trailed off towards zero as particles approached the (size) detection limit. In this study, the size distribution showed that MP were most abundant at the 36 µm major dimension, while nonsynthetic materials peaked at a somewhat larger particle size (47 µm). Such size distribution is not uncommon in microplastic studies^33^, and it is unclear what causes the trailing off of particle counts when approaching small particle sizes. First of all, it cannot be excluded that the measured size distributions reflect the true particle size distributions. On the other hand, it is possible that sampling, sample preparation, or sample analysis introduces a systematic error when sizes become small. In the present case, the sample preparation was probably not the cause, as it was limited to transferring particles from a silver filter to an IR-transmissive window without introducing any digestion steps as otherwise is common in MP studies. Neither was the sampling itself a likely cause, as it was conducted on a 0.8 µm pore size filter, and the probability of fibres slipping through was, hence, low. The analysis itself, though, might contribute to the phenomenon. The smaller a particle, the less IR light it absorbs, resulting in a poorer spectrum, which again will result in an increase in false-negative detections. Finally, surface forces might cause a higher tendency of entanglement for smaller particles compared to larger particles, leading to several agglomerated particles being identified as one. These phenomena might also have affected the counts and sizes for the natural particles.

It is interesting to note that the distribution at Location 3 had the smallest D50 values, and at the same time the highest MP exposure concentration, while Location 1 had the lowest MP concentration, but the highest D50 for the major dimension and the second highest for the minor dimension (Table 1). A linear regression using MP exposure concentrations and the relative D50 values highlights a negative correlation between the MP concentration and the median value of the correspondent size distributions. For increasing MP concentration, a decrease of D50 was observed (major dimension: R^2^ = 0.702; minor dimension: R^2^ = 0.735). This relation was limited to the MP particles, as no correlation between the nonsynthetic particles and the D50 value was found (major dimension: R^2^ < 0.001; minor dimension: R^2^ = 0.044). A further comparison between the size parameters of the MP and the nonsynthetic particles shows that the MP inhaled by the manikin tended to be smaller than the nonsynthetic particles. The major dimension D50 of the MP was 23% smaller than that of protein-based particles, and 53% smaller than that of the cellulose-based particles (Table 1). For the minor dimensions, the ratios were 32% and 40%, respectively.

The overall median value of the MP detected using this method (D50 = 36 µm) suggests that most of the particles inhaled are likely to undergo deposition by impaction, and therefore then eliminated by the mucociciliary escalator, so that a limited number is likely to reach the deeper airways^13,34^. Smaller fragments and fibres (<11 µm, sub-micrometric and nanometric particles) that can enter the lower airways may also have been present in the samples, but not detectable with the instrumental parameters used in this study. Although µFTIR-Imaging spectroscopy is a suitable technique for identifying particles potentially down to a few micrometres, further investigation is required to test if an enhanced analytical sensitivity (higher magnification, better resolution, better particle separation) could provide results for even smaller particles.

## Conclusion

The study showed that humans are exposed to indoor airborne microplastic pollution and that MP were ubiquitously present in the three investigated apartments. They comprised, on average, some 4% of all the inhaled organic particles identified, and were primarily of polyester. A comparison between the microplastic particles and the cellulose and protein-based material showed that particles of cellulose were as abundant as MP, but that the most abundant organic particles were of proteinous origin, probably shed skin. MP and nonsynthetic particles down to 11 µm (major dimension) could be identified, expanding the detectable size range reported by previous studies on air samples. The study showed that MP constitutes a non-negligible fraction of indoor airborne particulates, which can be inhaled and ingested. It also indicates that MP in inhaled air cannot be ruled out as having negative impacts on human health.

## Methods

### Sampling locations

The indoor air sampling was performed in three apartments in Aarhus, Denmark, during November and December 2017. The age and type of the buildings to which the apartments belong are different. The first apartment (Location 1) is part of a rather new lightweight building construction (2009), while the second and the third apartment (Location 2 and Location 3) are part of two typical Danish brick building constructions. All apartments consist of four rooms: a bedroom, a living room, a kitchen, and a bathroom. They are all naturally ventilated and equipped with conventional radiator heating systems (detailed information on the flats and their interior surfaces, like floors, walls, ceilings, is available in SI 2). Microplastic sampling was carried out for three consecutive days in each apartment, producing a total of nine samples, three per apartment. The residents carried out their normal day-to-day activities during the sampling. Due to the consecutive sampling, potential variations in the indoor environment could have occurred, and the samples were, hence, not replicates in a strict sense. However, the residents attempted to behave as similarly as possible from day to day, and a location-wise evaluation was, hence, carried out using the data collected from each apartment.

### Sampling device

**Figure 6:**
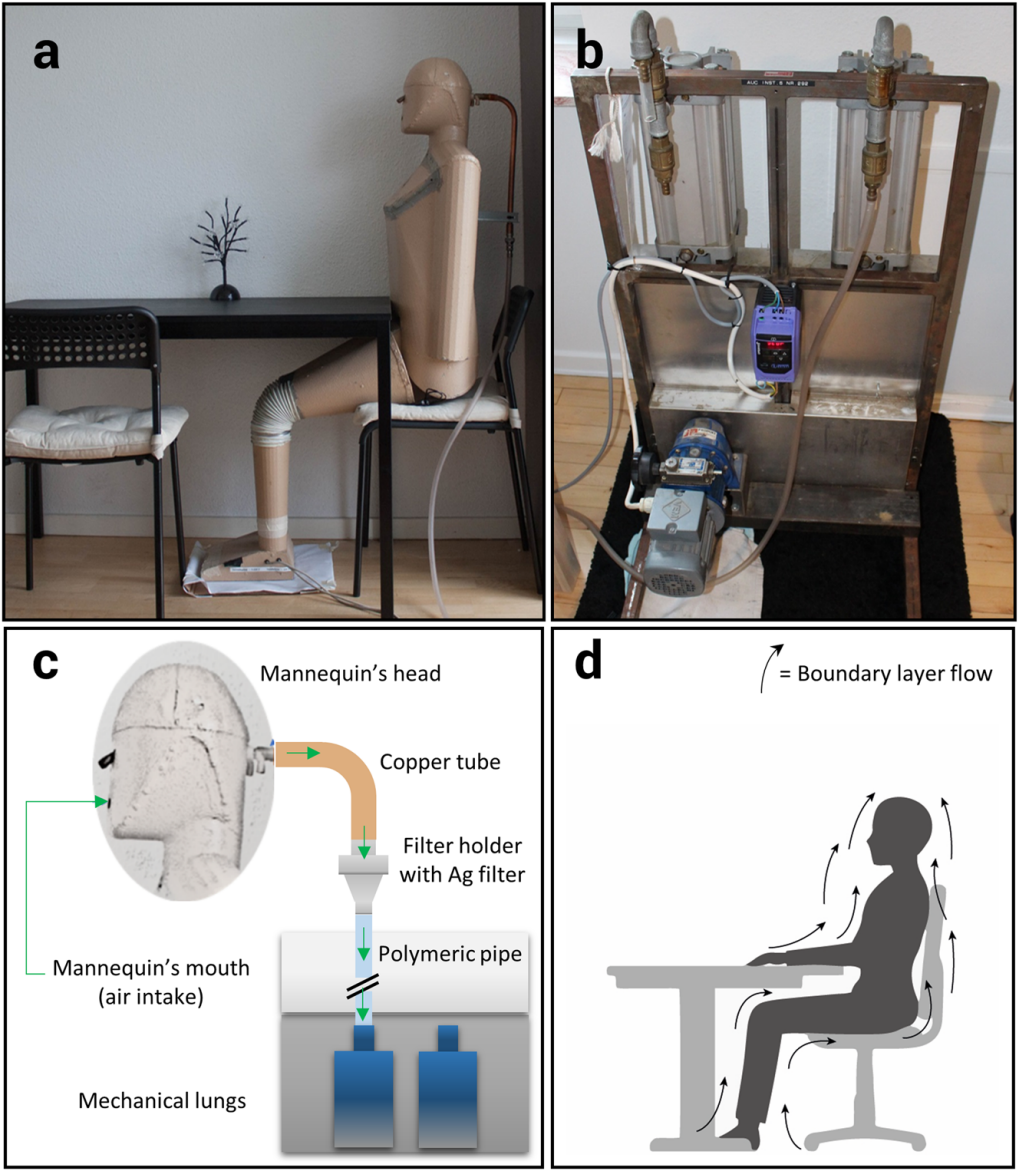
Sampling device setup: (**a**) manikin in seated position ready for sampling; (**b**) twin adjustable pistons connected to the motor to simulate breathing in and out; (**c**) sampling setup diagram; (**d**) Illustration of human boundary layer flow.

The sampling was conducted using a Breathing Thermal Manikin made of aluminium and glass fibre, having the purpose of simulating the presence of a person (Fig. 6). It was heated similar to a human body, creating a boundary layer flow around the manikin. The boundary layer is very important when measuring human exposure to room air pollution as most of the inhaled air is transported from lower regions of the room along the body to the mouth or nose^35–37^.

The measurements were performed in a sitting position at a table (around 110 cm)^38^ with a metabolic rate of 1.5 Met^39^ (105 W sensible heat)^40–42^ corresponding to light activity^43^, (SI 3). The manikin was connected to a mechanical artificial lung system, consisting of two pneumatic cylinders moved by an electric motor, producing an airflow simulating breathing. The volume of air released in each breathing cycle was determined by the piston stroke and breathing frequency (SI 3). A male respiration rate was chosen for this study, with a respiration frequency and volume flow of 14.26 min^−1^ and 0.82 l min^−1^, respectively. The sampling was carried out in periods of 24 hours, including periods with and without human activity, leading to a sampled air volume of 16.8 m^3^ per sample. The air samples were collected from the mannequin’s “mouth” air intake, which had an inner diameter of 9 mm.

### Filters and filter holder

A filter holder containing a filtering membrane was connected to the manikin (Fig. 6c). The inlet connection was made of a copper pipe, while the outlet pipe was a polymeric one. The filter holder was a steel/aluminium aerosol filter holder, with an active filtration surface of 133 mm^2^ (13 mm active diameter). The filters were 20 mm diameter custom-cut silver membranes of 0.8 µm pore size, obtained by tailoring 47 mm commercial filters (Sterlitech, Kent, WA, USA). The filters were flushed with nitrogen (N5.0) and stored in a pre-cleaned glass petri dish before sampling.

### Sample preparation

The silver filters could not be scanned directly, as a flat filter surface could not be maintained. An enriched membrane was hence submitted to a one-step sample preparation, transferring the sample to a more suitable support material. The filter was submitted to 5 minutes of sonication in a pre-cleaned small beaker filled with just enough ethanol (99.9%, HPLC grade, Chemsolute, Th. Geyer GmbH & Co, Germany) to cover the filter itself. The membrane was then flushed using an additional volume of ethanol, after which all the liquid containing the sample was deposited on a pre-heated (55 °C) zinc selenide (ZnSe) window held in a compression cell (PIKE technologies, Fitchburg, WI, USA) using a capillary glass pipette (micro-classic, Brand GmbH, Germany). The enriched ZnSe window was dried at 55 °C for 48 hours before submitting it to further analysis. The sample preparation was carried out in a lab equipped with an air filtration device (Dustbox® Hochleistungsluftreininger, Germany) with a HEPA filter (H14, 7.5 m^2^), and sample beakers were stored under glass protection during all sample preparation, as were the compression cells used for the final sample deposition. During scanning, the sample was protected from contamination by a collar which is an integrated part of the equipment, and the sample kept under a constant flow of pure nitrogen (N5.0). Three procedural blanks were prepared and analysed to evaluate potential contamination during the sample preparation and scanning process.

### FPA-µFTIR-Imaging

Sample analysis was carried out using FPA-µFTIR-Imaging spectroscopy(Focal Plane Array-Fourier Transform-Imaging-Micro-Spectroscopy), which is recognised as one of the most promising analytical techniques for the identification and quantification of microplastics^20–22,44,45^. The instrument was an Agilent 620 FTIR microscope equipped with a 128 × 128 pixel FPA detector, combined with a Cary 670 FTIR spectrometer (Agilent Technologies, Santa Clara, CA, USA). It produces two types of images: first, a magnified optical microscope image, and then an IR map made of stitched tiles of 128 × 128 pixels, co-adding several scans. The IR map of the sample contains an FTIR spectrum for each pixel, and allows the identification of a wide range of organic and inorganic materials comparing the unknown spectra with dedicated databases. This technique allows the rapid scanning of extended surfaces for material composition at very fine spatial resolution. The analysis was carried out by scanning the whole of each ZnSe window (active diameter of 10 mm, active area 78.5 mm^2^) in transmission mode, with an IR active range from 850 to 3750 cm^−1^. The following instrumental parameters were used: 128 × 128 FPA size; 15x IR Cassegrain objective-condenser system with 5.5 µm pixel size; 8 cm^−1^ spectral resolution; 30 co-added scans for each sample tile; 120 co-added scans for the background tile; beam attenuation 50%. The scan time was approximately 4 hours.

### Data analysis

Due to the high spatial resolution and the large area scanned, the amount of unbinned data produced per scan was around 22.5 GB or approx. 3.2 million spectra, leading to some challenges in the post-acquisition data handling. The software of the commonly used µFTIR-Imaging instruments struggle to manage such amount of data in a user-friendly way and they require substantial manual work by an analyst. This human interaction in the analytical flow furthermore introduces a bias into the analysis. In this study, a new software called MPhunter was applied to analyse the FPA data to automatically detect the particles on the scanned surface. MPhunter was developed at Aalborg University (AAU) in collaboration with Alfred Wegener Institut (AWI). The core of its particle identification is a correlation between the raw spectra, the 1^st^ derivative and the 2^nd^ derivative of all sample spectra to a custom-built spectral database containing more than 100 reference spectra (including polymers, paints, resins, and nonsynthetic materials). MPhunter is described in SI 1. The output from the automatic analysis was monitored and manually checked for possible false-positive and false-negative identification.

Particles were morphologically divided into fragments and fibres. There is no consensus in microplastics research on how to distinguish between the two shapes, and hence the definition applied by the World Health Organization^14^ for the determination of airborne fibres was adapted, where a fibre is defined as an object with a length to width ratio of >3. Fragments were consequently defined as objects having a length to width ratio of ≤3. The size limits were adapted to 11 µm for the major dimension and 5.5 µm for the minor dimension, as this was the lower limit of the applied settings of the FPA-µFTIR-Imaging. The major dimension of a particle was determined as the longest linear distance between pixels belonging to the particle. The minor dimension was calculated from the area of the particle, assuming it had an elliptical shape. Details on particle identification can be found in SI 1.

### Measuring particle exposure

Exposure refers to any contact between an airborne contaminant and a surface of the human body, either outer (for example the skin) or inner (for example the respiratory tract epithelium). Exposure is typically expressed quantitatively by a description of the duration of the contact and the relevant pollutant concentration^46^. In this study, MP’s simulated exposure was expressed as the concentration of MP inhaled by the manikin (number of MP per unit volume - N_MP_ m^−3^) during 24 hours of exposure. Protein-based and cellulose-based items (nonsynthetic) were also considered, and their concentration was expressed as done for MP (number of particles per unit volume – N_part_ m^−3^). Particle contamination was also considered at the three different locations, using the data obtained from the three measurements per location. The coefficient of variation calculated from each set of three consecutive measurements was considered to describe the intra-location variability. An overall exposure concentration was calculated from all samples.

### Particle size analysis

Particle size distribution analysis was carried out using the data provided by MPhunter based on the µFTIR-Imaging analysis (SI 1). Following previous studies^33,47^, the increment of the bins was chosen to 0.1 on a logarithmic scale. Besides the abundance of particles, the abundance of particles normalised by the size classes in micron was calculated, as well as the relative cumulative frequency^33^. D10, D50, and D90 values were calculated for each sample, location (sum of the particles identified from three samples collected at each location), and for the total number of identified particles. Size distributions were calculated using a particle’s area, and minor and major dimensions. The latter two parameters were used to distinguish between fibres and fragments, according to their length to width ratio.

### Statistical analysis

The normality of the datasets was tested by a Shapiro-Wilk normality test. An ANOVA plus Tukey HSD test, non-parametric ANOVA Kruskal-Wallis test, and Wilcoxon-Mann-Whitney test by rank was used to compare univariate groups in the datasets. All tests were performed using RStudio (v. 1.1.453).

## Supplementary information

Simulating human exposure to indoor airborne microplastics using a Breathing Thermal Manikin

## Data Availability

The datasets generated during and/or analysed during the current study are available from the corresponding author on reasonable request.
