# Supplementary material for: Simulating human exposure to indoor airborne microplastics using a Breathing Thermal Manikin
Source: Sci Rep. 2019 Jun 17;9:8670. doi: 10.1038/s41598-019-45054-w (PMC6573036; doi:10.1038/s41598-019-45054-w)
Supplement: Supplementary file 1 — Simulating human exposure to indoor airborne microplastics using a Breathing Thermal Manikin [file 41598_2019_45054_MOESM1_ESM.docx]

**Simulating human exposure to indoor airborne microplastics using a Breathing Thermal Manikin**

Alvise Vianello^1^*, Rasmus Lund Jensen^1^, Li Liu^2^, Jes Vollertsen^1^

^1^Department of Civil Engineering, Aalborg University, Thomas Manns Vej 23, 9220, Aalborg Øst, Denmark

^2^School of Architecture, Tsinghua University, Haidian District, 100084, Beijing, China

*Corresponding author e-mail: [av@civil.aau.dk](mailto:av@civil.aau.dk)

**Supplementary information (SI)**

**SI 1. Data analysis by MPhunter**

MPhunter^1^ is a freeware program developed at Aalborg University (AAU - Denmark) in collaboration with Alfred Wegener Institut (AWI - Germany), for automated detection of microplastics from µFTIR chemical imaging datasets. Such datasets make up a map of a scanned area where each pixel represents an IR spectrum. The maps can be created by a focal plane array (FPA) or by a linear array (LA), and the software can handle data files from all major vendors of µFTIR imaging systems. MPhunter compares each spectrum of an imaging dataset to each spectrum of a reference database and assigns a score to each such fit. It hence creates a set of maps of scores, where each pixel of a map represents the score to the respective reference spectrum. The score maps are then analysed to identify particles.

**Correlating µFTIR imaging spectra to reference spectra**

The algorithm that generates the score between a map spectrum and a reference spectrum compares the two raw spectra, their 1^st^ derivatives and their 2^nd^ derivatives. Figure 1 gives an example of such comparison for one pixel.


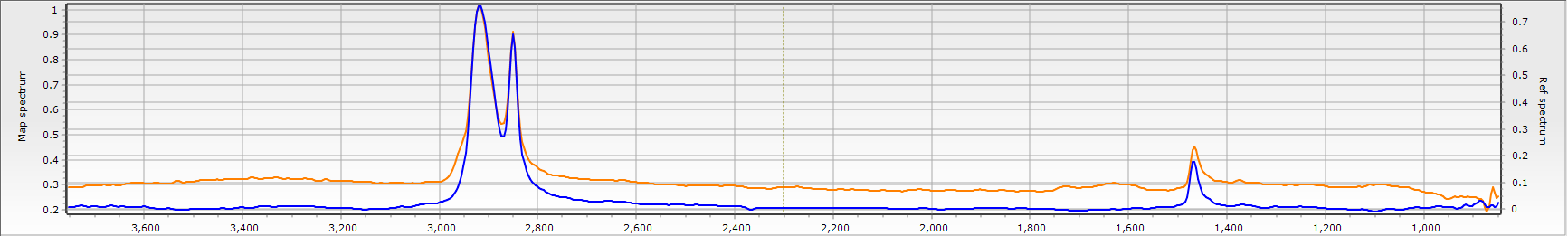


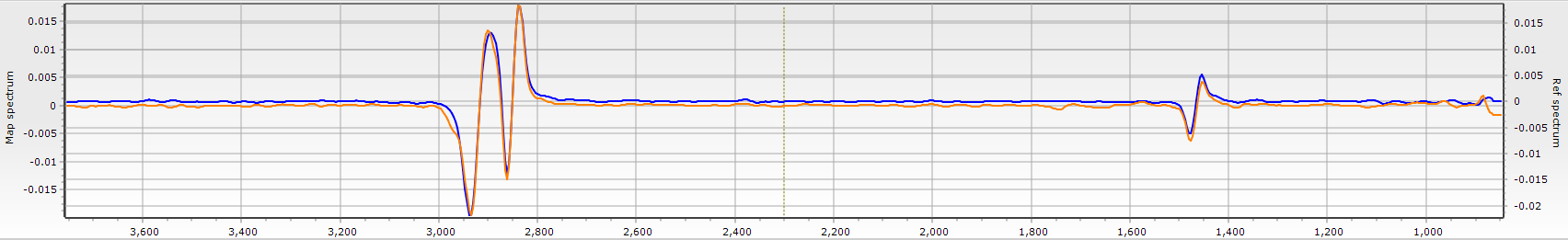


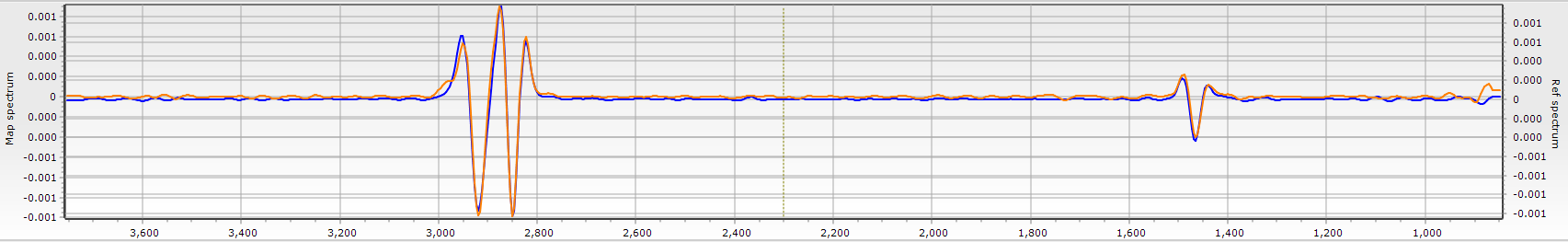


Figure 1. Comparison between a map spectrum (orange) and a reference spectrum (blue). The uppermost image is between the raw spectra, the middle image between the 1^st^ derivatives and the bottom image between the 2^nd^ derivatives.

The correlation between a reference spectrum and a map spectrum is done by a Pearson correlation, yielding 3 Pearson’s correlation coefficients (*r*) for each combination of map spectrum and reference spectrum: one for the raw spectra, one for the 1^st^ derivatives and one for the 2^nd^ derivatives (*r_0_*, *r_1_*, *r_2_*, respectively). The *r*-values are then squared and the user assigns global weights to the *r^2^*-values (*k_0_*, *k_1_*, *k_2_*, respectively). From this a score (*S_d_* where *d* refers to the spectrum in the reference database) is calculated for the fit between a reference spectrum and a map spectrum as:

$$S_{d}=\frac{k_{0}r_{0}^{2}+k_{1}r_{1}^{2}+k_{2}r_{2}^{2}}{k_{1}+k_{2}+k_{3}}$$

The score is hence always a value between 0 and 1. As default the program is set to omit negative correlations, i.e. if *r_0_*, *r_1_* or *r_2_* is < 0 then the respective *r_#_* is set to zero. Figure 2 gives an example of such correlation heat map, where a HDPE reference spectrum is correlated to spectra obtained from a 128 pixel FPA.


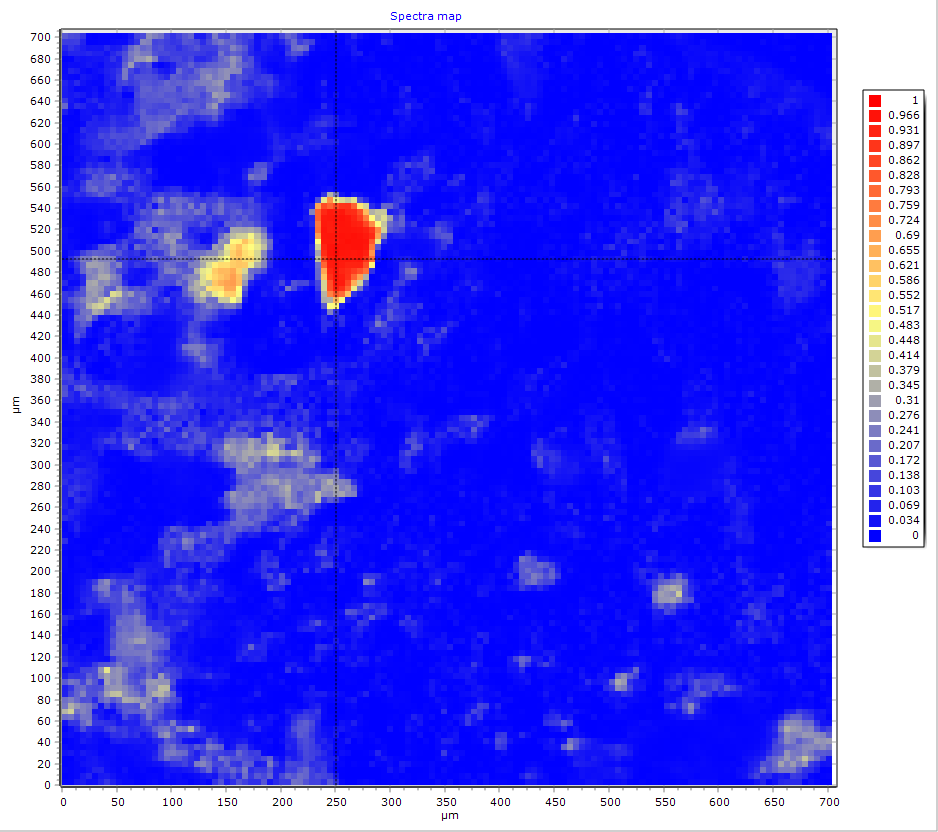


Figure 2. A heat map of correlations scores (S_d_) for a 128x128 pixels FPA image correlated to a HDPE reference spectrum (i.e. 128^2^ = 16,384 pixels). The pixel resolution of the FPA is set to 5.5 µm.

As default the program is set to ignore the CO_2_ absorption bandwidth (adjustable by the user). As an option the user can also narrow down the parts of the spectrum that are analysed, hence doing a correlation of selected parts of the spectra only. An example of such wavenumber range is shown in Figure 3.


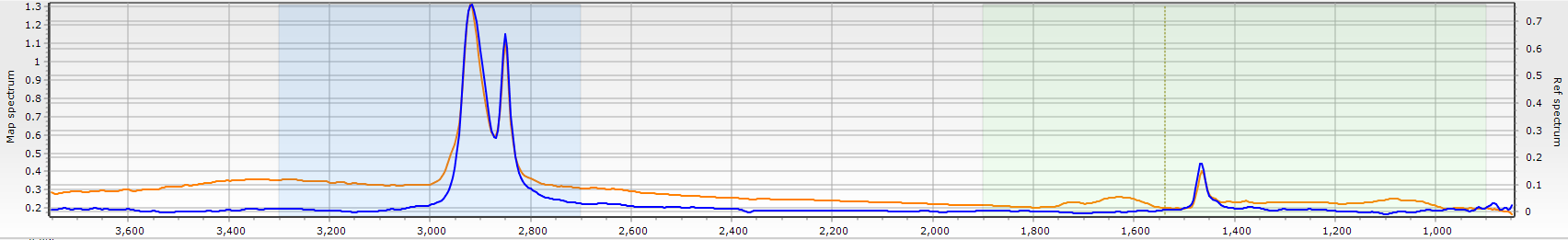


Figure 3. MPhunter allows as an option to correlation to two wavenumber ranges instead of the whole range.

**Automatic detection of particles of specific materials**

The analysis holds as many correlation maps as there are reference spectra in the database. These reference spectra will typically fall into fewer groups of materials, for example groups like PE, PP, PET, but also natural materials like cellulose and proteinaceous materials. When detecting plastic particles, it is adamant to include a range of natural materials because spectra of various natural materials show quite some similarities to those of the plastics. An example is proteinaceous materials and polyamide (nylon). The natural materials are hence used to reduce the risk of false positives in the analysis.

MPhunter allocates a particle to a material group through an algorithm based on score thresholds. The user sets 3 thresholds for each reference spectrum (*T_1_* > *T_2_* > *T_3_*). To qualify as a particle belonging to a material, at least one pixel must have a score of *S_d_ ≥ T_1_*. If several materials have a score above their respective *T_1_* value, then the pixel is associated to the material with the highest score. As an example, if a particle exceeds both the threshold of polyamide and proteinaceous materials, but the score for proteinaceous materials is higher than that for nylon, then that particle is associated with proteinaceous materials.

The pixel *S_d(i,j)_*, where (i,j) indicates the position on the map, is used as the nucleus of a particle. The algorithm develops the particle by looking at the adjacent pixels (Figure 4). If any of those have a score *S_d_ ≥ T_2_* for any reference spectrum belonging to the same material group as *S_d(i,j)_* then this pixel is associated to the particle created around *S_d(i,j)_*. After having developed the particle around *S_d(i,j)_*, the procedure is repeated around all pixels that have been added to the original particle nucleus.

Figure 4. A matrix of pixels around a pixel with a score S_r_ ≥ T_1_

In case there is an overlap between particles belonging to different material groups, and where the *S_d_* values in both cases are above the respective *T_2_* values, the pixel in question is associated to one of the particles according to the highest score. Such situation can for example arise when two particles of different materials overlap or are side-by-side in a scan.

In principle the above approach can give rise to particle sizes from one pixel and up. However, practical experience has shown that particles of only one pixel often are artefacts. The program was hence as default set to require at least 2 pixels to make up a particle. This value is user-adjustable.

The outcome of the detection is a set of discrete particles according to the material groups of the reference database. Figure 5 gives an example here of, where one image shows all particles and the other shows only microplastic particles. It is evident from the above that setting the thresholds is crucial for the outcome of the analysis. Practical experience with the algorithm, combined with a specialist knowledge on interpreting IR spectra, has been used to set individual thresholds for each reference spectrum. It is noteworthy that different materials require different thresholds to obtain a good hit as evaluated by manual interpretation of the map spectra. It shall also be noted that the thresholds should be re-evaluated for each new type of matrix, for different machines, and for different settings of a machine. For example, working with different magnifications does require different settings of the thresholds.


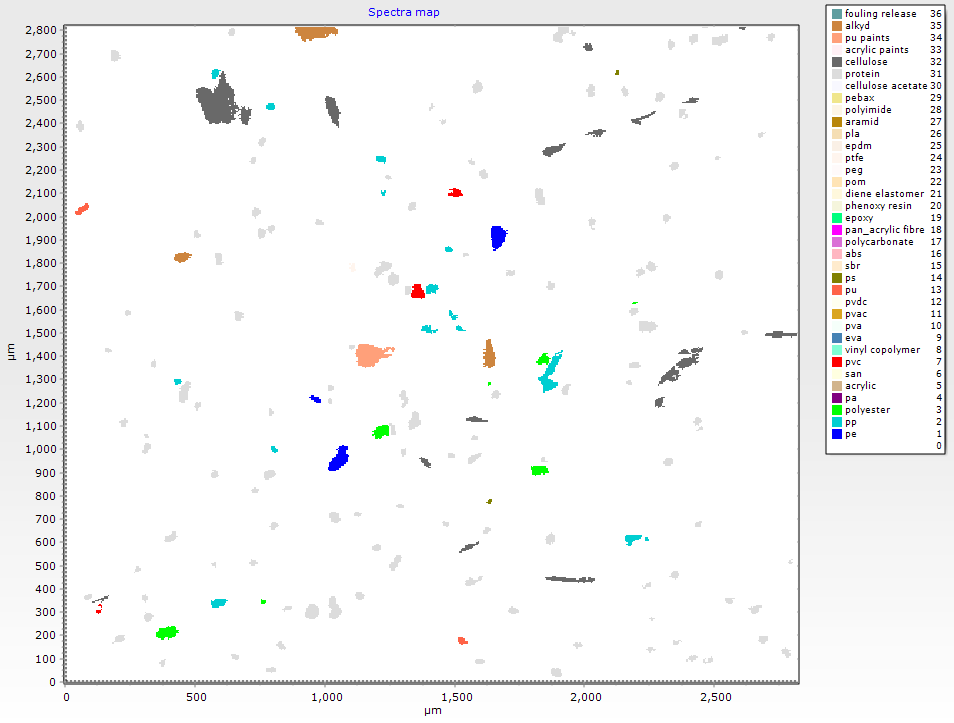

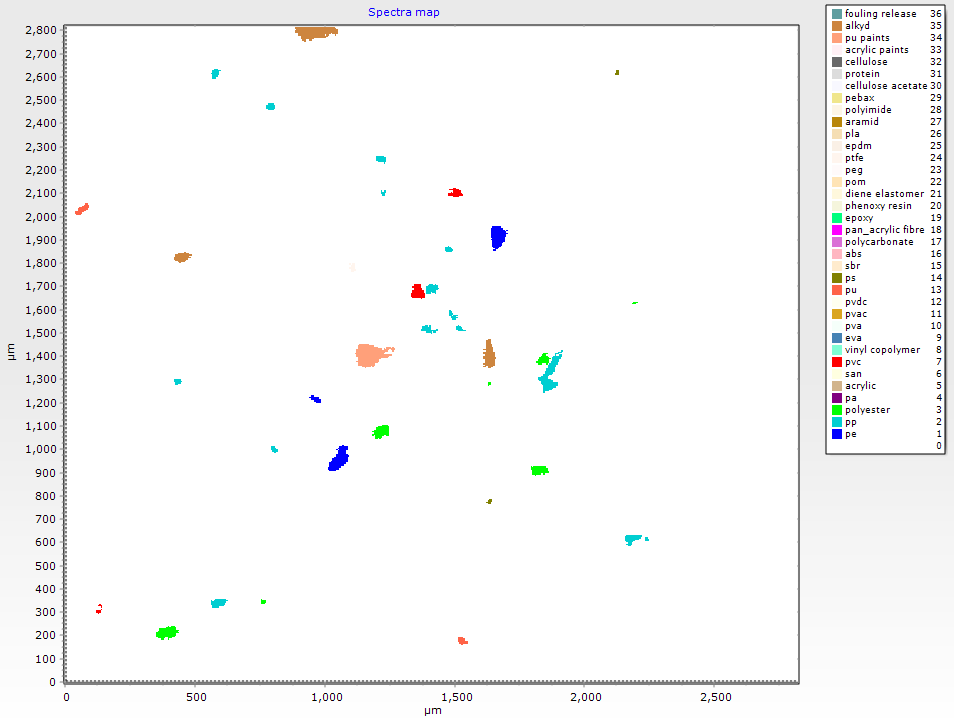


Figure 5. An image of an automated detection of particles. The left-hand image shows all detected particles, the right-hand image shows only microplastic particles. The scan consists of 512x512 pixels.

The user can choose to override the automatic detection of particles by adding and subtracting pixels from a particle, by joining particles and by deleting particles. Practical experience has shown that such manual verification can be minimized or even omitted if effort is put into setting the thresholds.

The third threshold, *T_3_*, is only used to limit the viewing of the list of scores shown for each pixel and is not involved in the analysis.

**Particle size and shape calculation**

The program calculates the major dimension of a particle by finding the longest distance between pixels of the particle. For particles consisting of <25 pixels, it is the single longest distance between any two pixels. For particles of 25-99 pixels, it is the average between the two longest distances, for 100-249 pixels the average of the three longest distances is used, and for particles of >250 pixels it is the average of the four longest distances which is used.

The minor dimension is derived from the area of the particle and its major dimension assuming that the particle has an elliptical shape. The particles are divided in two morphological categories according to their ratio length to width: fibre (elongated/fibrous particle) and fragments (non-elongated). All particles with a length to width ratio > 3 are assigned to the category “fibres”, while the particles with a ratio ≤ 3 are defined as fragments. This criterion is partially derived by the definition of a fibre given by the World Health Organisation, which defines a fibre as any particle with a length > 5 µm, width < 3 µm and aspect (length to width) ratio > 3:1^2^. Only the ratio is used as a criterion as the 3 µm width and 5 µm length thresholds are below the pixel resolution of the instrumental settings used for the FTIR-Imaging (5.5 µm). Using this approach, it is possible to distinguish two morphological categories using mathematical parameters and measure particle size with a good accuracy for simple shapes (Figure 5), as reported in Table 1 (a – d). However, this method has some limitations when complex shapes or bended fibres are measured such as the examples presented in Figure 6 and Table 1 (e – h).


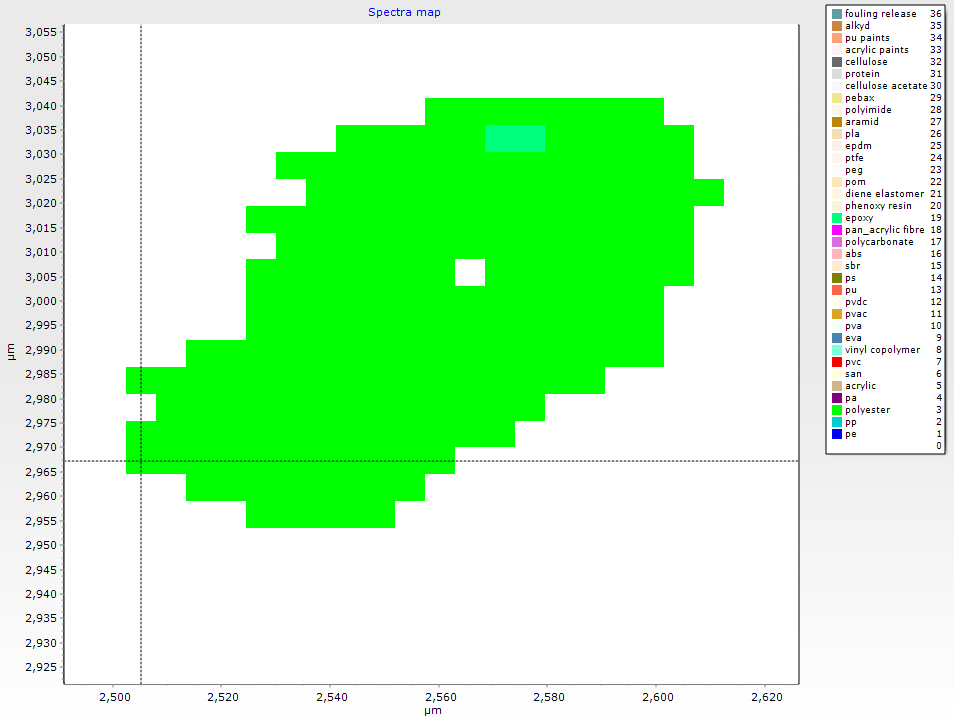

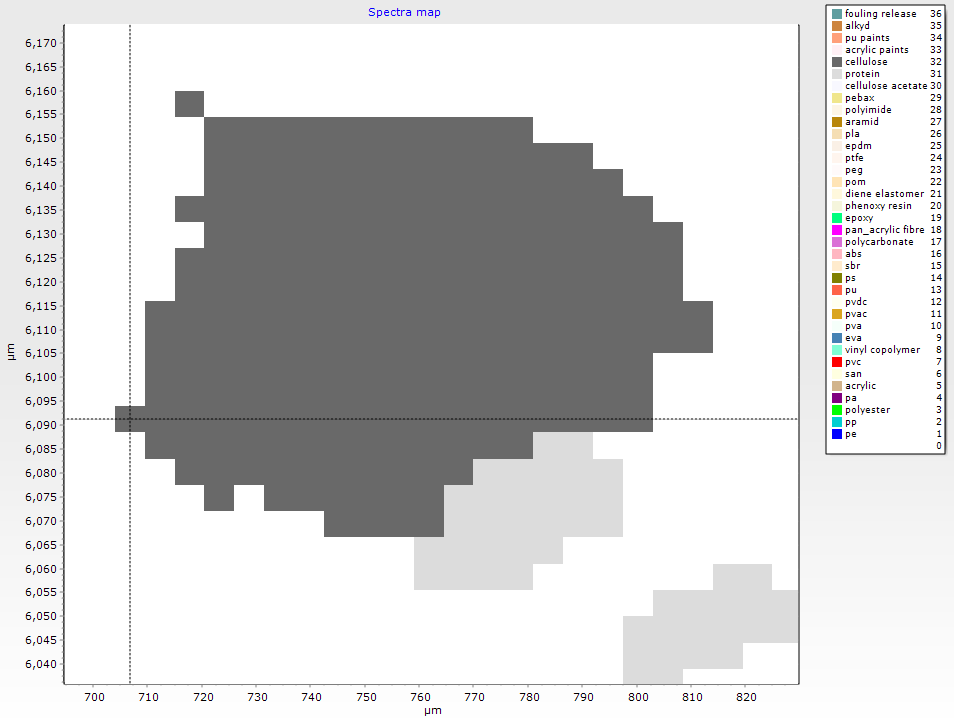

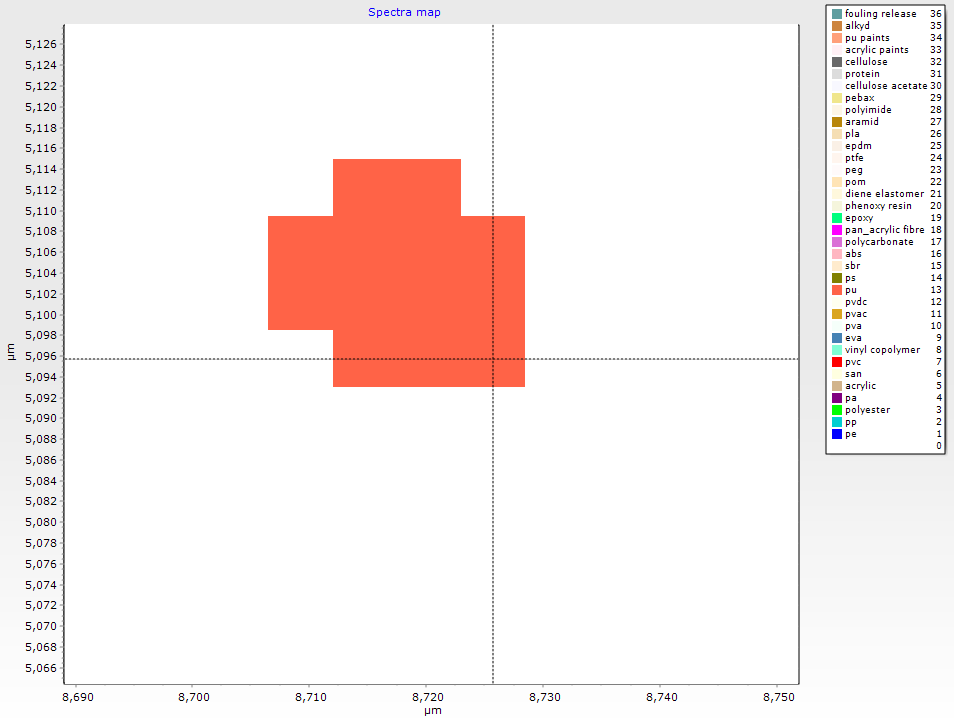

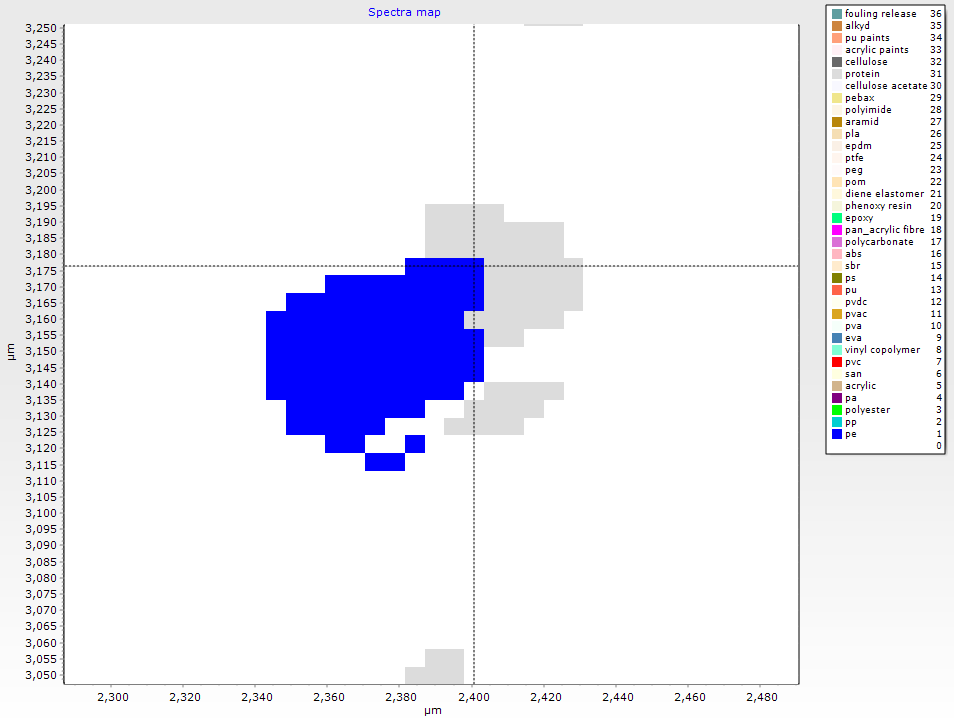


**d**

**b**

**a**

**c**

Figure 6. Four examples of fragments identified and measured using MPhunter.


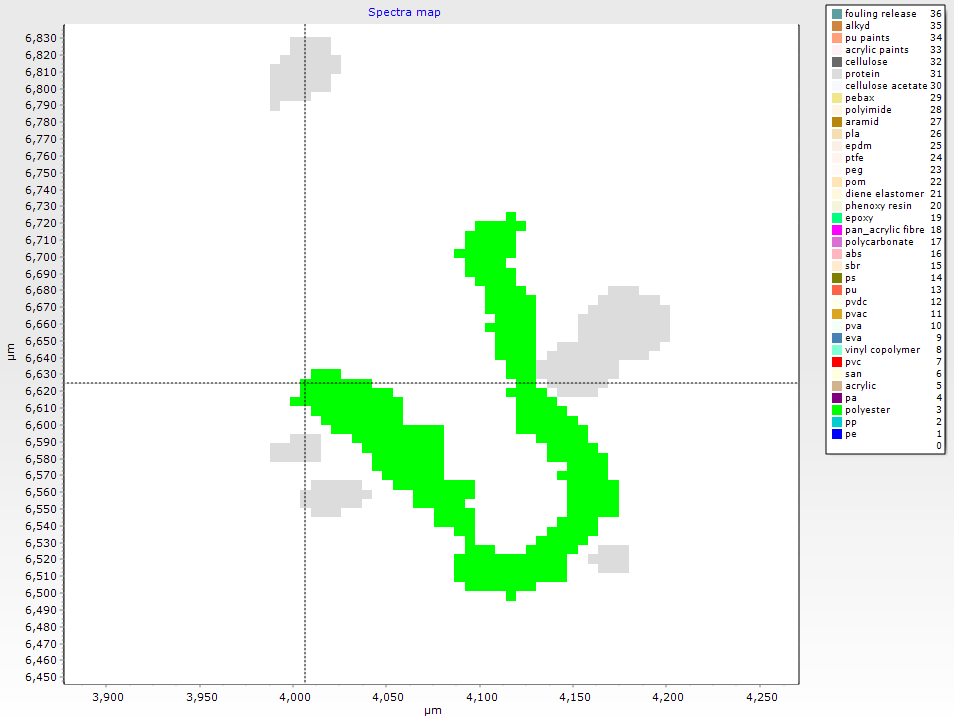

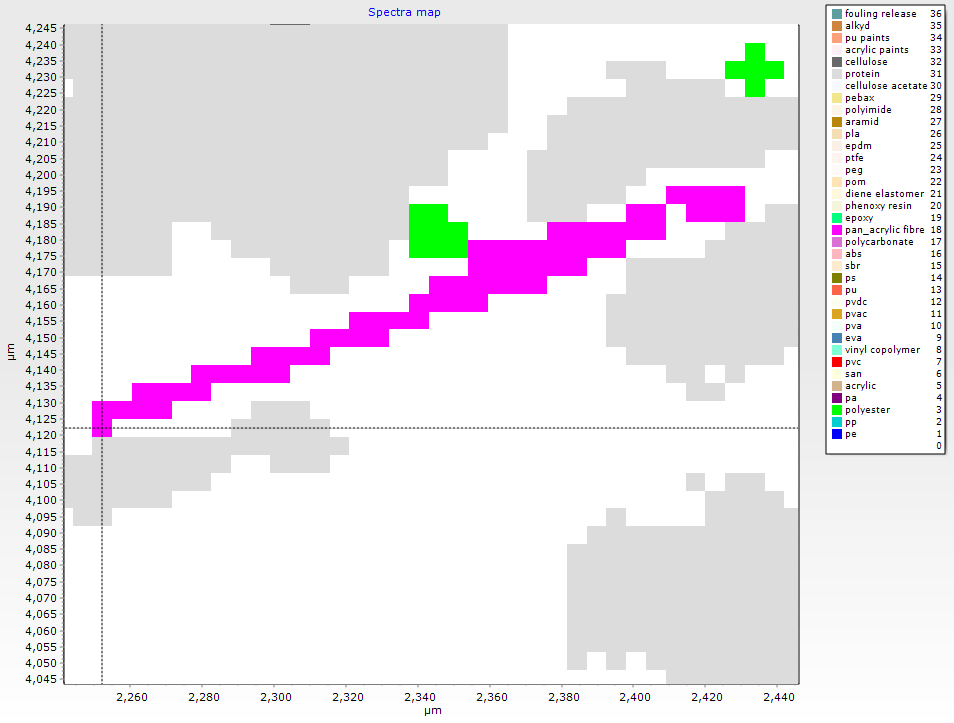

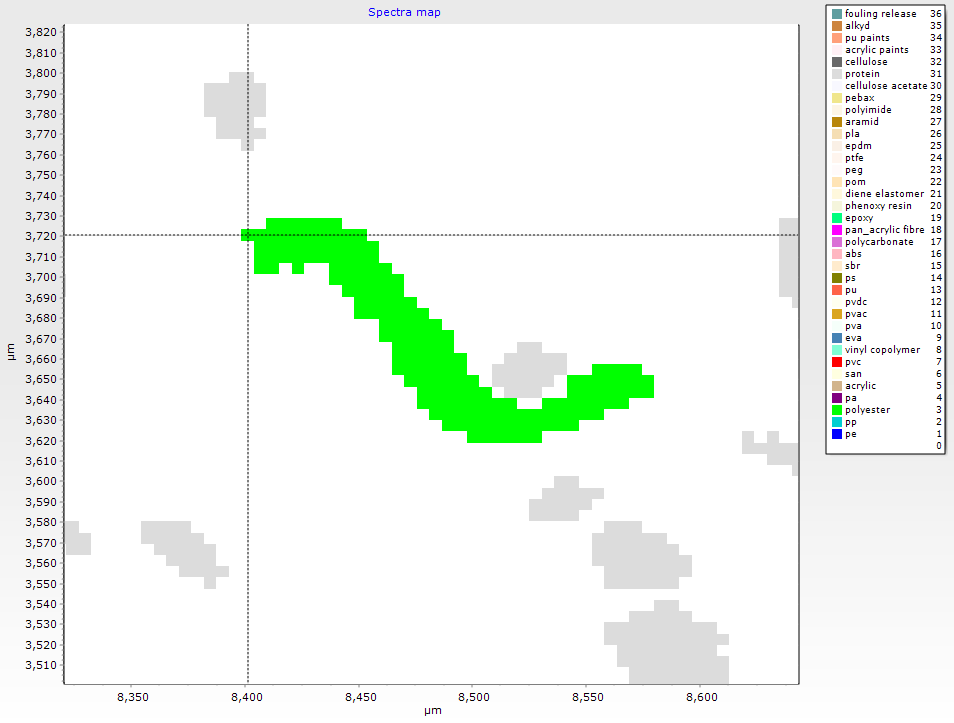

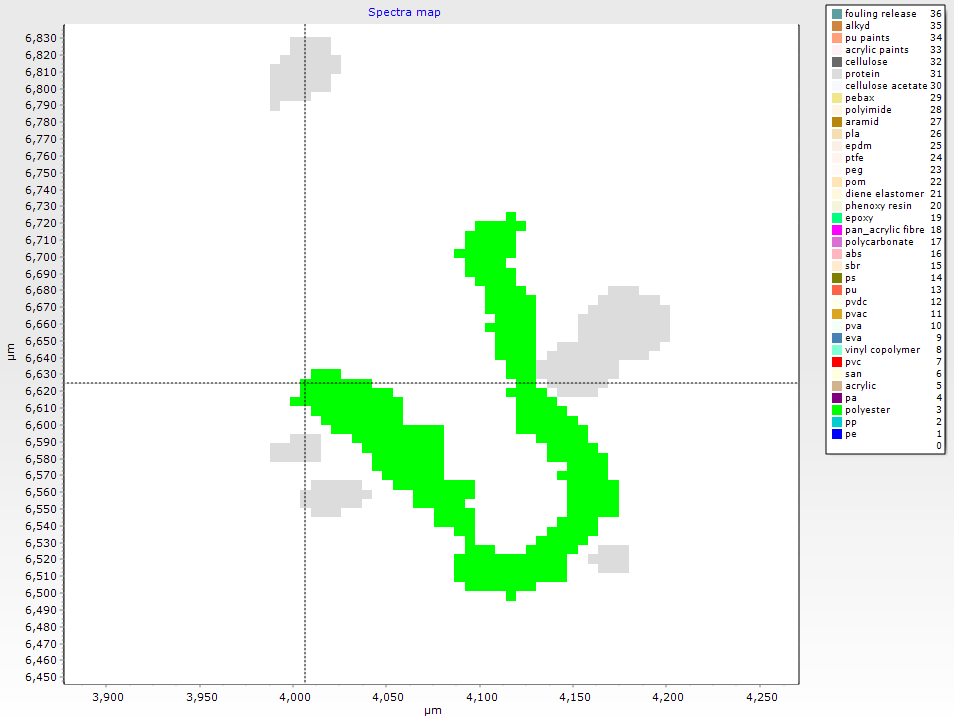

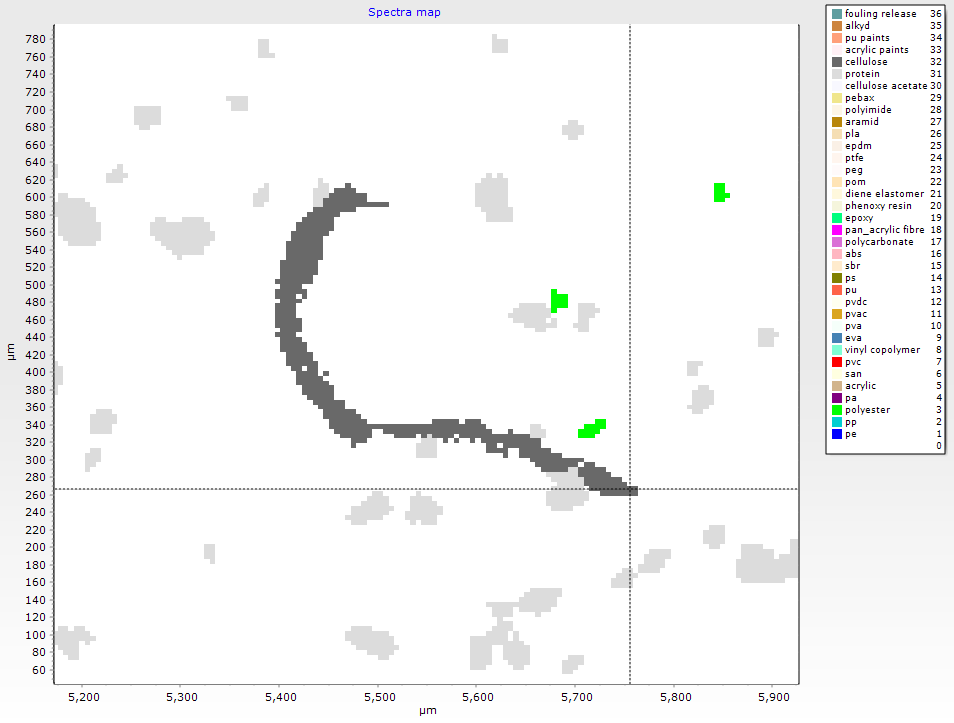


**h**

**f**

**e**

**g**

Figure 7. Four examples of fibres identified and measured using MPhunter. e) and g) represent a straight and semi-straight fibre, while f) and h) are bended fibres, representing a complex shaped particle.

| Particle ID | Material | Area (µm2) | Auto Major dimension (µm) | Manual Major dimension (µm) | Auto minor dimension (µm) | Manual minor dimension (µm) | Ratio Auto measurement | Ratio Manual measurement |
| --- | --- | --- | --- | --- | --- | --- | --- | --- |
| a | Polyester | 6020 | 119 | 129 | 62 | 73 | 1.9 | 1.8 |
| b | Cellulose | 6927 | 112 | 112 | 79 | 104 | 1.4 | 1.1 |
| c | PU | 393 | 25 | 25 | 19 | 21 | 1.4 | 1.2 |
| d | PE | 2783 | 74 | 76 | 48 | 50 | 1.6 | 1.5 |
| e | PAN | 1966 | 195 | 196 | 13 | 15 | 15 | 13 |
| f | Polyester | 10194 | 228 | 399 | 57 | 33 | 4 | 12 |
| g | Polyester | 5143 | 195 | 232 | 34 | 29 | 6 | 8 |
| h | Cellulose | 14944 | 465 | 655 | 41 | 35 | 11 | 19 |

*Table 1. Main automatic and manual size parameters related to the fragments and fibres displayed in Figure 5 and Figure 6, respectively.*

While the automatic size determination is generally good for fragments, it does decrease if the fibre is bent. To test the performance of the automatic measurement, fifty particles with a “fragment-like” shape and filthy with “fibrous-like” shape were manually measured using a measuring tool in MPhunter following the centreline of the shape of the particle. The width was measured in triplicate, in the central area and close to the ends of the particle, and the mean value was used for further comparison. The differences between the automatic and manual measurements are displayed in Figure 7.


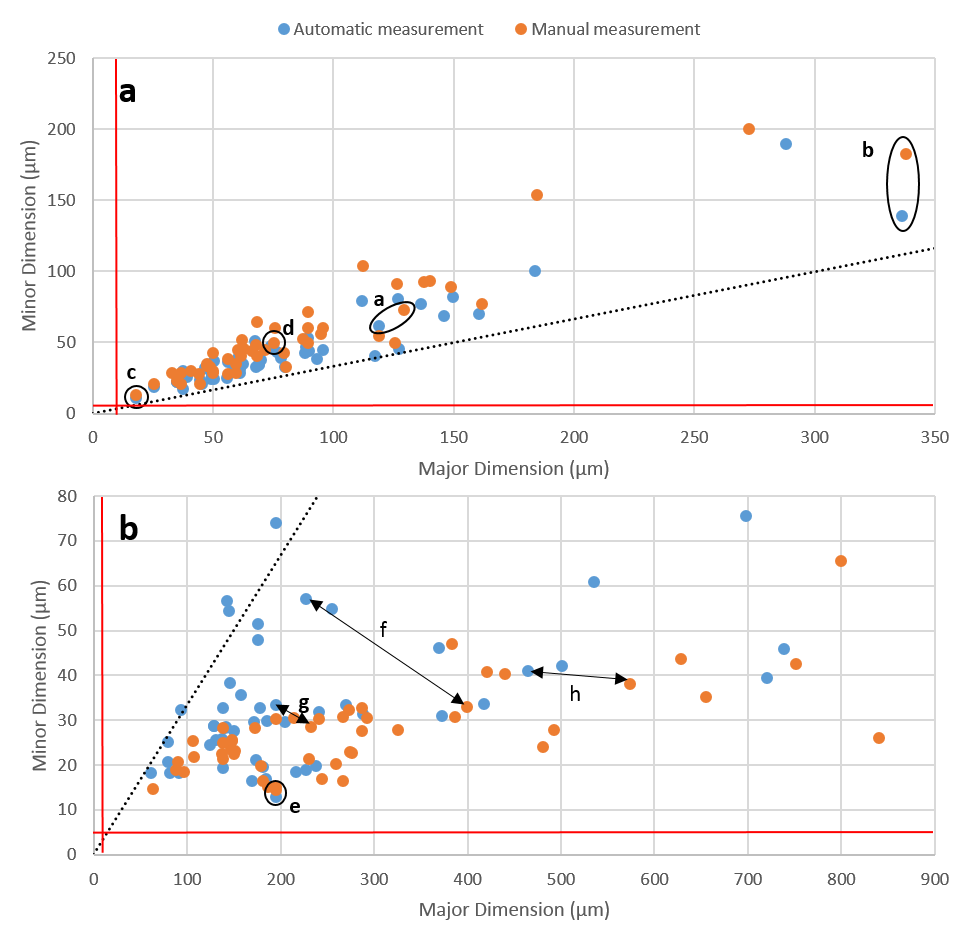


Figure 8. The minor dimension plotted against the major dimension for manual and automatic measurements of fragment-like (a) and fibrous-like (b) particles (N = 50 for both sub-samples). The dashed line indicates the threshold for fibre assignment (ratio 3:1; y = x/3). The red vertical and horizontal lines indicate the Limit of Detection of the technique in term of size for major (11 µm) and minor (5.5 µm) dimension (2x1 pixels). The four fragments (a, b, c, d) and the four fibres (e, f, g, h) of Figure 5 and 6 are highlighted on the plot, showing the difference between automatic and manual measurement.

The main statistics for the automatic and manual measurements are reported in Table 2 and 3 for the fragments-like and fibre-like and particles, respectively. All the distributions were non-normal distributed, both for the fragment-like and fibre-like particles (Shapiro-Wilk test, p < 0.05). Regarding the fragment-like sub-sample, the automatic and manual measurements were not significantly different for neither the major nor the minor dimension (p = 0.986 and p = 0.092, respectively; Mann-Whitney-Wilcoxon test). The distributions obtained by the ratio major:minor dimension were though significantly different (p = 3.274e^-5^; Mann-Whitney-Wilcoxon test). Looking at the fibre-like particles, automatic and manual measurements were not significantly different for the major dimension (p = 0.102; Mann-Whitney-Wilcoxon test), while the two distributions for the minor dimension differed significantly (p = 3.49e^-06^; Mann-Whitney-Wilcoxon test). The distributions obtained using the ratio major:minor dimension resulted in differences for manual and automatic measurements (p = 2.053e^-07^; Mann-Whitney-Wilcoxon test).

|  | **Min** | **D10** | **Q1** | **Q2 (D50)** | **Mean** | **Q3** | **D90** | **Max** | **Std** |
| --- | --- | --- | --- | --- | --- | --- | --- | --- | --- |
| **Area** | 151 | 714 | 1097 | 1921 | 4437 | 3630 | 8343 | 42955 | 7879 |
| **Auto_Major** | 18 | 38 | 50 | 68 | 86 | 95 | 146 | 337 | 60 |
| **Manual_Major** | 18 | 37 | 50 | 68 | 86 | 96 | 141 | 338 | 59 |
| **Auto_minor** | 11 | 22 | 28 | 37 | 46 | 48 | 79 | 190 | 31 |
| **Manual_minor** | 13 | 27 | 31 | 44 | 55 | 60 | 93 | 200 | 38 |
| **Ratio_AM** | 1 | 1 | 2 | 2 | 2 | 2 | 2 | 3 | 0 |
| **Ratio_MM** | 1 | 1 | 1 | 2 | 2 | 2 | 2 | 3 | 0 |

*Table 2. Main statistics for the automatic and manual measurements of the selected fragments.*

|  | **Min** | **D10** | **Q1** | **Q2 (D50)** | **Mean** | **Q3** | **D90** | **Max** | **Std** |
| --- | --- | --- | --- | --- | --- | --- | --- | --- | --- |
| **Area** | 877 | 2783 | 2783 | 4159 | 7028 | 7124 | 7124 | 41382 | 7770 |
| **Auto_Major** | 61 | 139 | 139 | 177 | 232 | 240 | 240 | 739 | 163 |
| **Manual_Major** | 64 | 149 | 149 | 237 | 287 | 369 | 369 | 840 | 190 |
| **Auto_minor** | 13 | 22 | 22 | 30 | 34 | 41 | 41 | 76 | 15 |
| **Manual_minor** | 15 | 21 | 21 | 26 | 27 | 31 | 31 | 66 | 10 |
| **Ratio_AM** | 3 | 4 | 4 | 6 | 7 | 9 | 9 | 18 | 4 |
| **Ratio_MM** | 4 | 6 | 6 | 9 | 10 | 12 | 12 | 32 | 5 |

Table 3. Main statistics for the automatic and manual measurements of the selected fibrous particles.

According to the sub-sample of fragment-like particles used (N = 50), the median value for automatic and manual measurements resulted in the same value for the major dimension (68 µm), while the median value for the minor dimension (automatic measurements) was 16% smaller the one obtained for the manual measurements. The median value of the ratio major:minor dimension for the automatic and manual measurements gave the same value (2). Regarding the sub-sample of fibre-like particles (N = 50), the median value for automatic measurements was 12% smaller for the major dimension and 13% larger minor dimension than the manual measurements. As a consequence the median value of the ratio major:minor dimension for the automatic measurement was 60% of the median value of the same ratio for the manual measurements. As a result, the fraction composed by elongated particles can have been underestimated in favour of the non-elongated particles, especially when complex and bended shapes are numerous in a sample, or when the bias is related to particles close to a ratio of 3.

**SI 2. Detailed information of the sampling locations**

**GPS coordinates of the sampling locations and their position on the map**


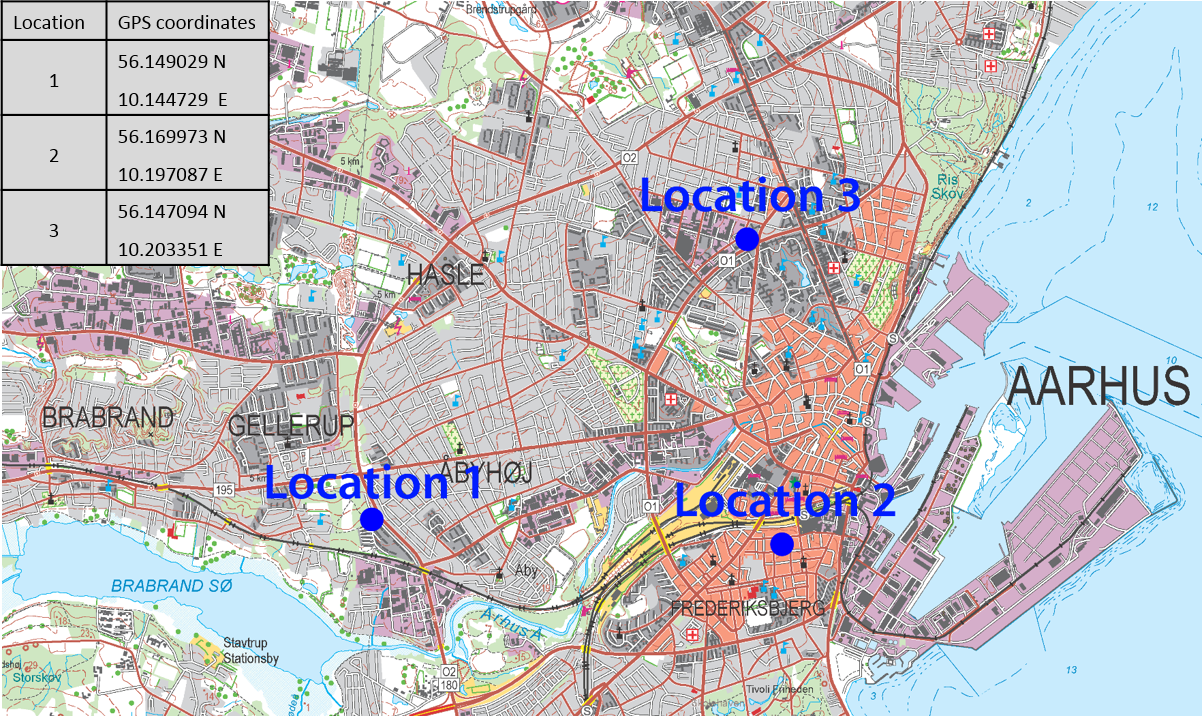


Map of the city of Aarhus, Denmark. Sampling locations are highlighted with blue circles and the relative GPS coordinates are reported in the merged table. The map contains free data from Styrelsen for Dataforsyning og Effektivisering, K100_2017^2^.

**Description of the sampling locations**

**Location 1**

| **Address** | Aabyhøjgård 40, 8230 Aarhus |
| --- | --- |
| **GPS coordinates** | 56.149029N – 10.144729E |
| **Year of construction** | 2009 |
| **Building type/Materials** | Lightweight; steel structural frame. |
| **Surface** | 50 m^2^ |
| **N of rooms** | 4 |
| **Ventilation** | Natural; mechanical extraction in the bathroom and in the kitchen |
| **Heating system** | Radiators; floor heating in the bathroom |
| 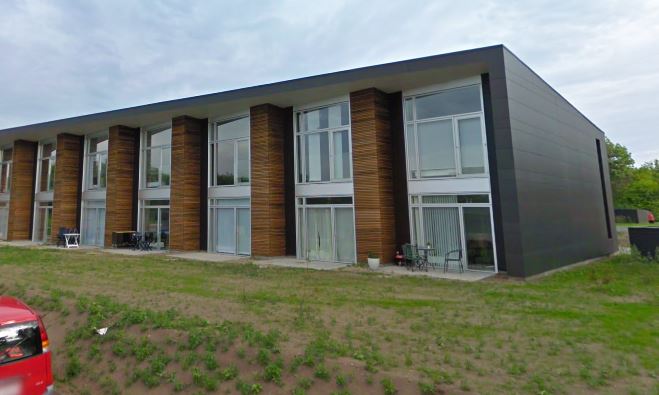**External facade** | **Map of the apartment**  **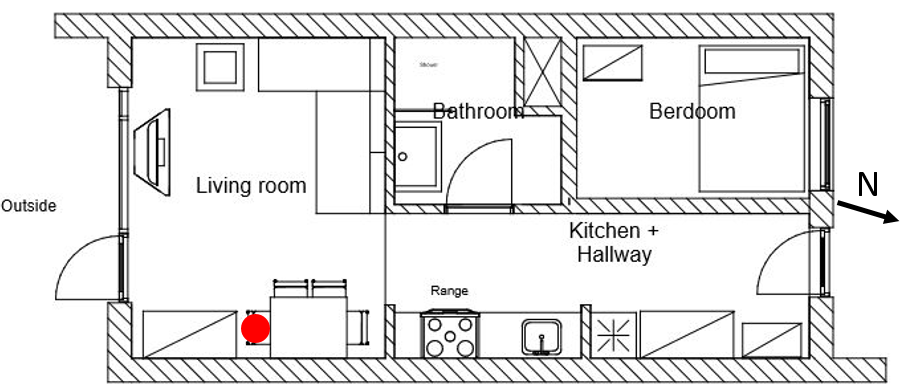**  **BTM position** |
| **Picture of the room** | **Materials of the main surfaces** |
| 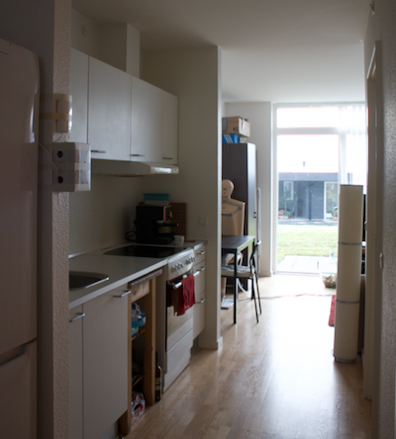 | **Hallway & Kitchen**  Surfaces:  Floor - Wooden Parquet  Walls – Wall Paper + White Paint  Ceiling – Plaster + White Paint |
| 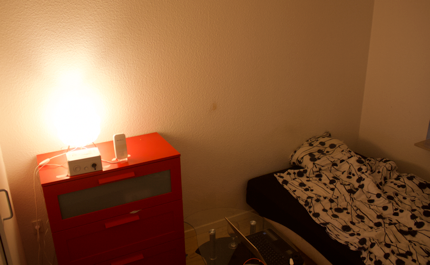 | **Bedroom**  Surfaces:  Floor - Wooden Parquet + Small Carpet  Walls - Wall Paper + White Paint  Ceiling - Plaster + White Paint |
| 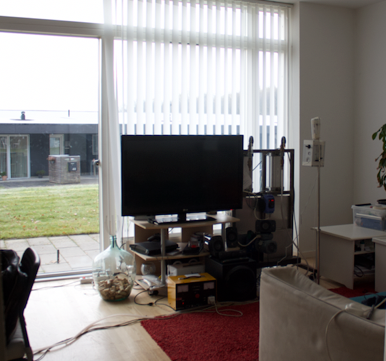 | **Living-Room**  Surfaces:  Floor - Wooden Parquet + Small and Big Carpet  Walls - Wall Paper + White Paint  Ceiling - Plaster + White Paint |
| 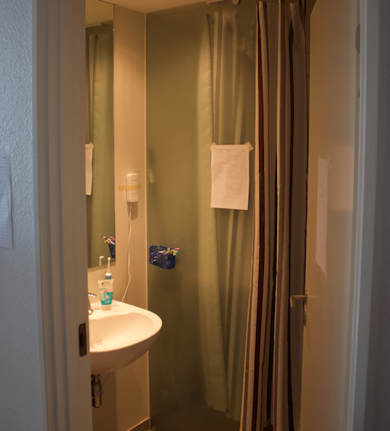 | **Bathroom**  Surfaces:  Floor – Tiles  Walls - Plaster + White Paint Moisture Resistant  Ceiling - Plaster + White Paint Moisture Resistance |

**Location 2**

| **Address** | Brammersgade 12, 8000 Aarhus C |
| --- | --- |
| **GPS coordinates** | 56.169973N – 10.197087E |
| **Year of construction** | Originally 1897, renovated a couple of times |
| **Building type/Materials** | Solid brick construction |
| **Surface** | 53 m^2^ |
| **N of rooms** | 4 |
| **Ventilation** | Natural; mechanical extraction in the bathroom (activated by light) and in the kitchen (exhaust hood) |
| **Heating system** | Radiators with common thermostatic valve |
| **Number of inhabitants** | 2 |
| **External facade**  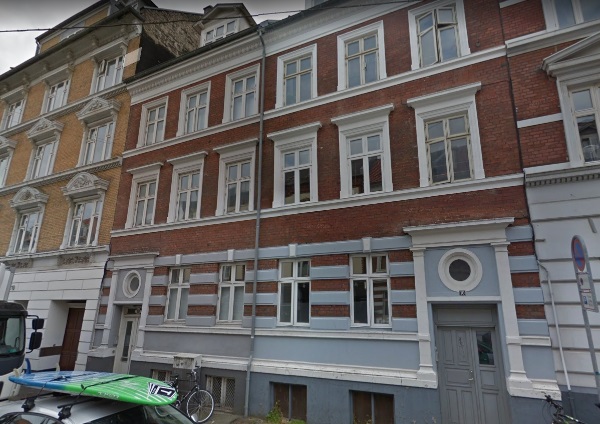 | **Map of the apartment**  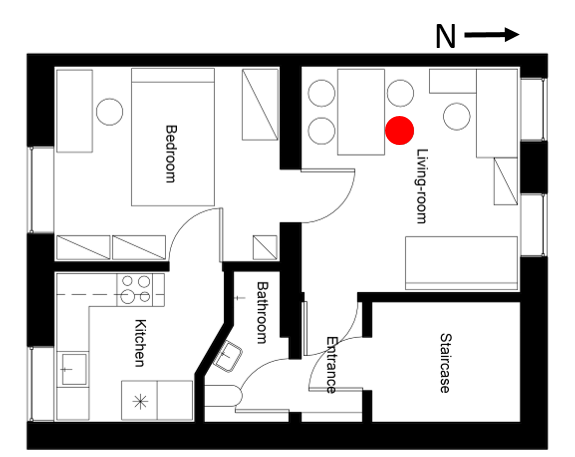  **BTM position** |
| **Picture of the room** | **Materials of the main surfaces** |
| 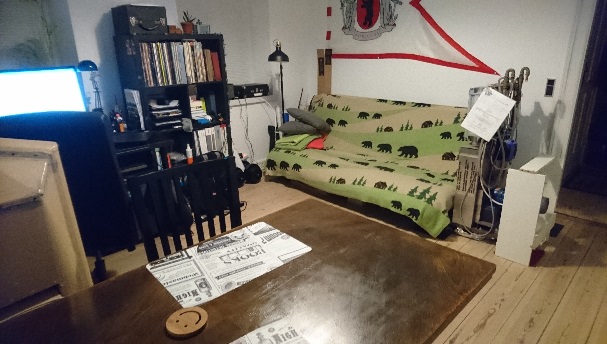 | **Living-room**  Surfaces:  Floor – Wood boards  Walls – Solid brick construction, plaster, wallpaper painted white  Ceiling – Plaster, painted white |
| 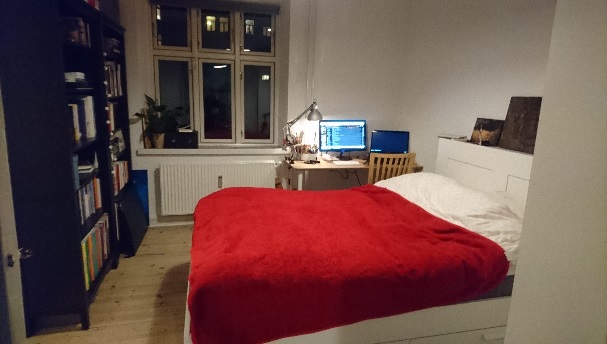 | **Bedroom**  Surfaces:  Floor – Wood boards  Walls – Solid brick construction, plaster, wallpaper painted white  Ceiling – Plaster, painted white |
| 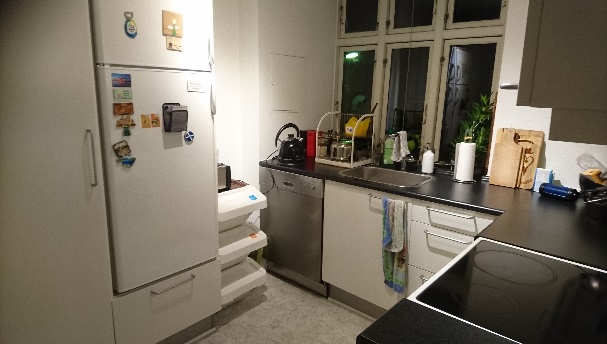 | **Kitchen**  Surfaces:  Floor – Linoleum  Walls – Solid brick construction, plaster, wallpaper painted white  Ceiling – Suspended ceiling, a single layer of plasterboard painted white. |
| 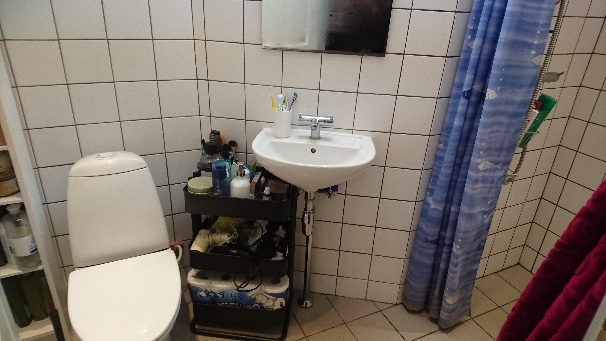 | **Bathroom**  Surfaces:  Floor – Concrete, wet room membrane, tiles  Walls – Solid brick construction, wet room membrane, tiles  Ceiling – suspended ceiling, a single layer of plasterboard painted white. |

**Location 3:**

| **Address** | Venstre Ringgade 230, 8000 Aarhus C |
| --- | --- |
| **GPS coordinates** | 56.147094N – 10.203351E |
| **Year of construction** | 1947 on an older layout (middle 1800’s) |
| **Building type/Materials** | Solid brick walls and timber joints as the floor partitions |
| **Surface** | 55 m^2^ |
| **N of rooms** | 4 (plus 1 hallway) |
| **Ventilation** | Natural; mechanical extraction in the bathroom (activated by light an relative humidity) and in the kitchen (exhaust hood) |
| **Heating system** | Radiators with common thermostatic valve |
| **Number of inhabitants** | 2 (12-24/11/2017); 3 (24/11 – 02/12/2017); 4 (02-05/12/2017) |
| **External facade**  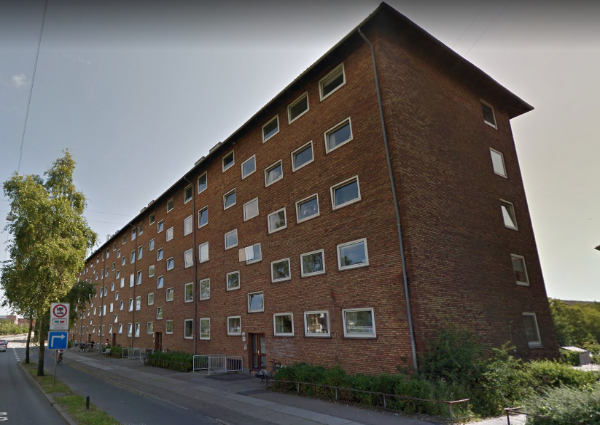 | **Map of the apartment**  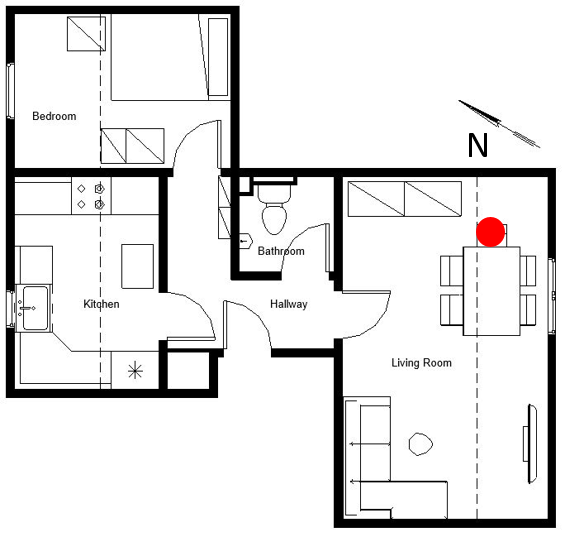  **BTM position** |
| **Picture of the room** | **Materials of the main surfaces** |
| 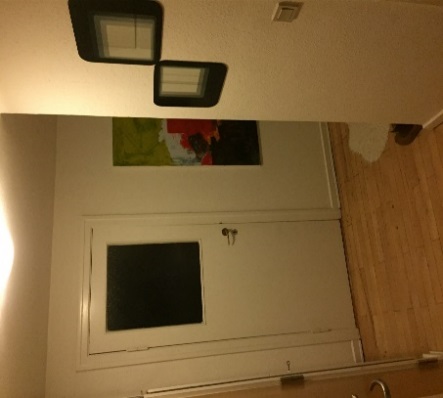 | **Hallway**  Surfaces:  Floor - Wooden Parquet  Walls – Wall Paper + White Paint  Ceiling – Plaster + White Paint |
| 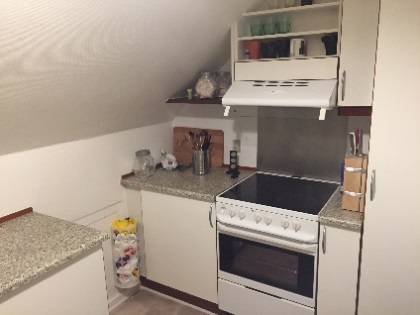 | **Kitchen**  Surfaces:  Floor – Vinyl Surface  Walls – Wall Paper + White Paint  Ceiling – Plaster + White Paint |
| 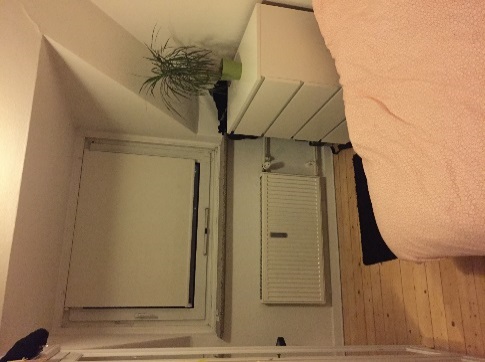 | **Bedroom**  Surfaces:  Floor - Wooden Parquet + Small Carpet  Walls - Wall Paper + White Paint  Ceiling - Plaster + White Paint |
| 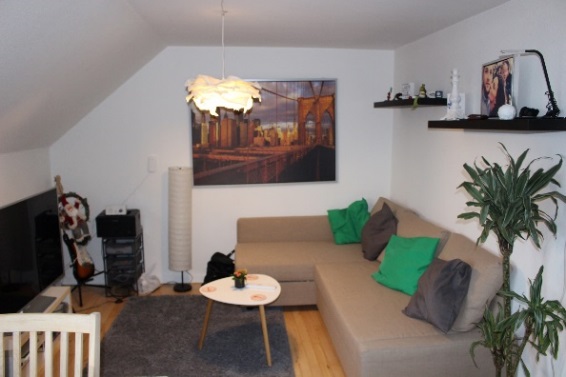 | **Living-Room**  Surfaces:  Floor - Wooden Parquet + Small and Big Carpet  Walls - Wall Paper + White Paint  Ceiling - Plaster + White Paint |
| 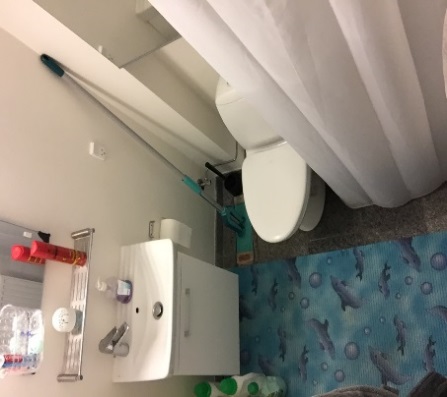 | **Bathroom**  Surfaces:  Floor – Tiles + Small Synthetic Cover  Walls - Plaster + White Paint Moisture Resistant  Ceiling - Plaster + White Paint Moisture Resistance |

**SI 3. Description of the sampling equipment**

The Breathing Thermal Manikin (BTM), (Figure 6), is a female size manikin, made out of aluminium and glass fibre, having the purpose of simulating the presence of a person. It is able to release heat and simulate breathing in and out, depending on the desired metabolic rate. For the measurements, in a sitting position with light activity, the metabolic rate was set at 1.2 Met or the equivalent of approximately 105 W of sensible heat. The manikin is hollow and heated by an ajustable internal heating element. To ensure an evenly distribution of heat and thus a representative skin temperature the air is mixed by a fan throughout the mannequin’s internal space.

The lungs consist of two pneumatic cylinders which are moved by a motor. The movement of the pistons creates a flow of air which is directly linked to the size of the cylinder, speed of the motor and stroke length of the piston. Therefore, the volume flow and the breathing frequency can be adjusted.

As there is a difference between the male and female respiration rates and also a variation due to activity level it was decided to use a respiration frequency and volume flow of 14.26 min^-1^ and 0,82 L min^-1^. This corresponds to 16.8 m^3^ per 24 hours.

**References**

1. Liu, F., Olesen, K. B., Borregaard, A. R. & Vollertsen, J. Microplastics in urban and highway stormwater retention ponds. *Sci. Total Environ.* **671**, 992–1000 (2019).

2. Organization, W. H. Determination of airborne fibre number concentrations: a recommended method, by phase-contrast optical microscopy (membrane filter method). 1–53 (1997). doi:ISBN 92 4 154496 1

1. Styrelsen for Dataforsyning og Effektivisering, K100 2017. Data downloaded 11/20/2018.
